# A Review of Binderless Polycrystalline Diamonds: Focus on the High-Pressure–High-Temperature Sintering Process

**DOI:** 10.3390/ma15062198

**Published:** 2022-03-16

**Authors:** Jérémy Guignard, Mythili Prakasam, Alain Largeteau

**Affiliations:** UMR 5026, ICMCB, CNRS, Universite Bordeaux, F-33600 Pessac, France

**Keywords:** diamonds, binderless, synthesis, sintering, high pressure, high temperature, physical properties

## Abstract

Nowadays, synthetic diamonds are easy to fabricate industrially, and a wide range of methods were developed during the last century. Among them, the high-pressure–high-temperature (HP–HT) process is the most used to prepare diamond compacts for cutting or drilling applications. However, these diamond compacts contain binder, limiting their mechanical and optical properties and their substantial uses. Binderless diamond compacts were synthesized more recently, and important developments were made to optimize the P–T conditions of sintering. Resulting sintered compacts had mechanical and optical properties at least equivalent to that of natural single crystal and higher than that of binder-containing sintered compacts, offering a huge potential market. However, pressure–temperature (P–T) conditions to sinter such bodies remain too high for an industrial transfer, making this the next challenge to be accomplished. This review gives an overview of natural diamond formation and the main experimental techniques that are used to synthesize and/or sinter diamond powders and compact objects. The focus of this review is the HP–HT process, especially for the synthesis and sintering of binderless diamonds. P–T conditions of the formation and exceptional properties of such objects are discussed and compared with classic binder-diamonds objects and with natural single-crystal diamonds. Finally, the question of an industrial transfer is asked and outlooks related to this are proposed.

## 1. Introduction

Carbon (C) is the fourth most abundant element in the universe and is mainly produced in massive stars during thermonuclear fusion of three He atoms. On Earth, carbon is the fifteenth most abundant element in crust; however, its total estimate it still under debate [1] due to its ubiquity in higher atmosphere layers to inner core under various forms.

Indeed, carbon can be present as organic (e.g., oil, coal), inorganic (CO_2_, CO) and pure (C) components. Under its pure form, only three natural structures are reported: graphite, lonsdaleite and diamond, whereas a lot of synthetic carbon phases have been created over the last decades, e.g., graphene, fullerene, nanotubes, glassy carbon, amorphous carbon, Q-carbon and others.

Due to its electronic structure of 1s^2^2s^2^2p^2^, pure carbon can adopt sp2 and sp3 hybridization, conferring the possibility to be bonded with three or four neighbors, respectively. Hence, graphite (sp2)–diamond (sp3) phase transition, without any catalyst, needs a very high activation energy in the form of high pressure and high temperature in the orders of 10 GPa and several hundred degrees, respectively, compared to the transition line. This difference in hybridization type also explains the huge difference in their physical properties.

Diamonds have always fascinated people and scientists, first because natural diamonds are rare and are used as luxury products. In spite of being such a simple component, natural diamonds offer the best mechanical and optical properties and very advanced thermal and electrical properties. Therefore, scientists have put a lot of effort into reproducing diamonds in the laboratory since the late nineteenth century. All these efforts have produced a lot of technical developments in high-pressure research, with a wide range of applications in Earth and material science [2,3,4]. Additionally, these efforts have produced a new desire in diamond geological settings as well as in mineral physics to determine and understand this phase transition.

The goal of this review is two-fold: (i) provide a brief overview of natural and synthetic diamonds as well as their properties and uses and (ii) provide a state of the art of synthesis/sintering techniques of binderless diamonds under high-pressure–high-temperature (HP–HT) conditions. These synthetic diamond compacts have received a lot of attention in the last two decades since the first success of direct conversion sintering (DCS) of graphite at 20 GPa and T > 2000 °C [5]. Indeed, these new synthetic diamonds offer a wide range of applications based on their exceptional mechanical, optical and electrical properties that can surpass those of classical polycrystalline diamond compact (PDC) and even those of natural diamonds. Therefore, the focus of this review will be on technical improvements over the decades as well as on the research of material with ultimate properties, in more reasonable conditions, in order to consider a potential industrial transfer.

## 2. The Different Forms of Pure Carbon

### 2.1. Natural

**Figure 1:**
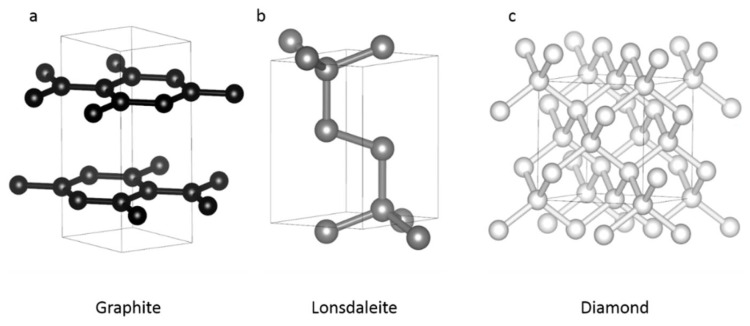
Crystal structures of the three natural forms of carbon. From left (**a**) to right (**c**), i.e., with increasing pressure and temperature, (**a**) graphite (black), (**b**) lonsdaleite (dark grey) and (**c**) diamond (light grey). Graphite and lonsdaleite have hexagonal structure, whereas diamond is cubic. Graphite has a sp2 hybridization, whereas lonsdaleite and diamond have an sp3 one.

In nature, pure carbon exists only under three different crystalline structures: graphite, which is stable at ambient pressure and temperature, and two high-pressure–high-temperature minerals (lonsdaleite and diamond) (Figure 1, Table 1).

Graphite has a hexagonal structure (Table 1, space group # 194, a = b= 2.456 Å, c = 6.696 Å, [6]) and is formed by a staking of layers composed of regular hexagons of carbon (Figure 1a). Each atom of a given layer is covalently bonded to three others, making an sp^2^ hybridization type, whereas between layers, bonds are weaker and could be of Van der Waals type. This would be the reason for the low hardness of graphite (1–2 on Mohs scale).

Lattice structure of lonsdaleite (also called hexagonal-diamond) is also hexagonal (Table 1, space group # 194, a = 2.51 Å, c = 4.12 Å, [7]). This kind of intermediate structure between graphite and diamond has an sp3 hybridization (Figure 1b). This form of carbon was discovered in Canyon Diablo (Arizona) meteor crater. Lonsdaleite is very rare and mostly found in meteorites and craters [8], even though it was also reported in ultra-high-pressure context on Earth [9]. It is therefore a high-pressure high temperature form of carbon but its stability field, if any, is still poorly constrained [10]. Main difference with diamond is the stacking of atoms, with an AB–AB sequence. Properties of lonsdaleite are also debated, and there are discrepancies between measured and calculated [11] physical properties, such as for density (3.3 and 3.52 g/cm^3^), hardness (7–8 and 12 on Mohs scale) and indentation resistance (predicted to be 152 GPa). If these properties were confirmed, lonsdaleite would be harder than diamond.

Diamond, the hardest known phase among natural and synthetic materials, has a face-centered cubic lattice (Table 1, space group # 227, a = 3.56679 Å, [6]). In this compact structure (ρ = 3.51 g/cm^3^), atoms are covalently bonded with three others with an average bond length of ~1.54 Å, due to sp3 orbitals hybridization (Figure 1c). Hence, atoms form tetrahedral structure (Figure 1c) confer incredible physical properties to this mineral. Stacking sequence is of ABC–ABC–ABC type, making a complex suite of polytypes between lonsdaleite (2H) and diamond (3C). Diamond stability field covers a wide range of P–T conditions that can be enlarged due its metastability field.

### 2.2. Synthetic

High-purity carbon forms were fabricated over the last decades with their own properties, and several hundred allotropes were predicted through calculation. A lot of reviews were focused on this topic, including synthetic and predicted forms ([12] and reference therein). This section will focus on the brief description of common synthetic forms of carbons and those that are also commonly used (other than graphite) as starting materials for the synthesis and/or sintering of high-pressure phases such lonsdaleite and diamonds. In most cases, these precursors are either highly disordered or contain already a certain amount of sp3 bonds. These two features could play a role to decrease the P–T conditions of formation of diamonds compared to graphite (high order, only sp2 bonds). More details on this respect are given in Section 5.

***Graphene*** corresponds to a mono-layer of graphite, constituting a 2D structure of hexagons. In graphene sheets, each atom of carbon is covalently bonded to its three nearest neighbors. Graphene is known for its exceptional electronic (high electric conductivity), optical, thermal (high conductivity) and mechanical (highest young modulus) properties.

***Pyrolytic carbon*** is a synthetic form of carbon, close to graphite, produced by the pyrolysis of hydrocarbon. The main difference with graphite is the presence of covalent bonds between graphene layers, making pyrolytic carbon a highly orientated structure with unusual physical properties, e.g., higher thermal conductivity than graphite, and magnetic levitation. Pyrolytic carbon is mainly used for military, plastics, metallurgy, electronic and medicine.

***Fullerenes and carbon nanotubes*** (CNTs) have similar bonds to graphene but are 3D bodies with spherical and cylindrical shapes, respectively. In these structure, carbon atoms and bonds form, in addition to the classic hexagons, pentagons and/or heptagons that prevent a 2D structure as in graphene. These two compounds form two big families, with more or less complex structure, e.g., from C20 to C70 for fullerenes and from single *(SWCNTs)* to multi-wall nanotubes *(MWCNTs)*. In most cases, they were fabricated by vaporization of carbon at high temperature and neutral atmosphere (He or Ar). Nanotubes were also synthesized using CVD method. Due to their physical and chemical properties, fullerenes and nanotubes are the second most used nanoparticles, after silver [13]. Fullerene applications are widespread, including for pharmacy, medicine, cosmetics and electronics [14]. Applications of *CNTs* are more recent and are still under development but are already used as composite fibers in polymers [15,16] and could be also promising in electronics [17,18], environment [19] and medicine [20]. It has to be noted that fullerene exists in nature, particularly in space. However, it was first synthesized in lab before being identified as fullerene in space.

***Amorphous carbon*** (aC) is a carbon allotrope that does not have a crystalline structure [21]. In mineralogy, this term is used for coal that is, in reality, a mixture of very fine polycrystalline graphite. For the past few decades, it has been possible to synthesize thin films of *aC* using CVD [22] or arc deposition [23] methods. Atomic bond lengths and distances are unique compared to all other carbon allotropes. According to the synthesis methods, the ratio sp2-sp3 hybridization can be controlled. Applications are wide and concern textiles, healthcare, water filtering and food packaging [24]. More recently, a new form of *aC* was synthesized by a process of surfusion: the Q-Carbon *(QC),* which seems to exhibit physical properties at least similar to those of diamonds [25,26].

***Glassy- (or vitreous) carbon*** (GC) is a non-graphitizing, non-graphitizable form of carbon that is fabricated through the carbonization of polymeric precursors at high temperature (>1200 °C). Staking of disorientated graphite layers, a huge disordered structure and a high level of closed micro-porosity, are the main “lattice” features of *GC*, conferring a low density of 1.5 g/cm^3^. GC has unique properties, e.g., high temperature and chemical resistance, impermeability to gases and liquid, low friction and low electrical resistance. *GC* is mainly used as electrode in electrochemistry and as crucible in high-temperature experiments.

Other precursors can be used to synthesize high-pressure phases of carbon. However, they are organic compounds such as polycyclic aromatic hydrocarbons (PAH), stearic acid, diamondoids, methane (CH_4_) and even explosives charges (Trinitrotoluene TNT or Hexogen RDX).

## 3. Natural Diamonds: Geological Settings and Classification

### 3.1. Natural Diamonds: Some Geological Settings

Natural diamonds occur in various sources such as Earth mantle [27,28], crater impact [29,30], meteorites [31] and even interstellar environment [32], as evidenced by diamond presolar grains.

**Figure 2:**
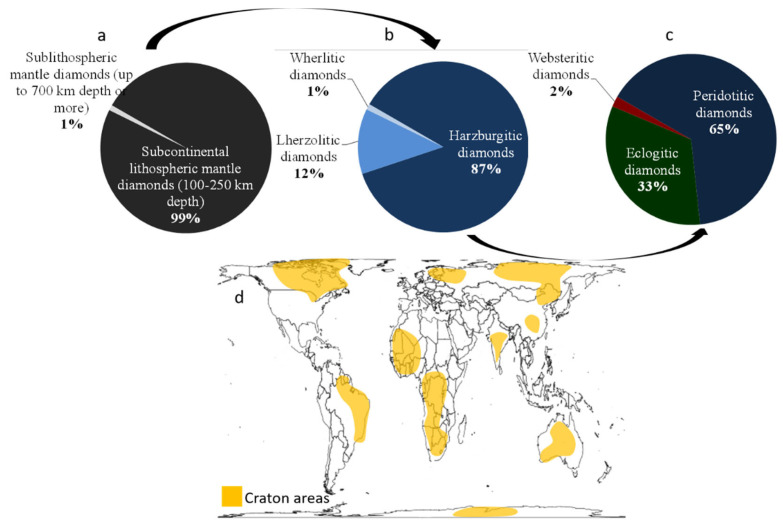
Main geological settings for the formation of natural diamonds. (**a**) Different setting as a function of depth of formation. (**b**) Geological settings for the lithospheric mantle diamonds. (**c**) Parageneses observed for the most common peridotitic diamonds. (**d**) World map for the localization of the main cratons on Earth. These cratons are the main diamond deposits.

Conditions of formation of natural diamonds on Earth are very well documented and mainly based on mineralogy and chemistry of diamond inclusions. It has been shown that 99% of diamonds originated from subcontinental lithospheric mantle (up to 200–250 km depth), with the last percent corresponding to a deeper rare origin in the sublithospheric mantle (up to at least 700 km depth) (Figure 2a). In the former case; i.e., lithospheric diamonds, three groups can be distinguished: peridotitic (65%), eclogitic (33%) and websteritic (2%) diamonds ([33], Figure 2b). Among the peridotitic suite, another three-part subdivision, according to the paragenese, was also made between harzburgitic (56% of all natural diamonds), lherzolitic (8%) and wherlitic (0.7%) diamonds origin ([33], Figure 2c). Based on the geothermobarometry of minerals constituting diamond inclusions, pressure–temperature conditions of diamond formations were estimated around 5–6 GPa and 1000–1200 °C, with few variations according to their environment of formation [33].

Hence, diamonds could form from various mechanisms such as (i) direct conversion through the graphite–diamond phase transition at relevant conditions, (ii) precipitation from a fluid or melt saturated with carbon and (iii) oxidation-reduction reactions involving methane and/or carbonates. The first mechanism is the most unlikely as it has been shown experimentally that an overpressure of at least 10 GPa is needed to directly transform graphite to diamond (>15 GPa and ~2000 °C, [5]), conditions that are far from natural conditions of formation. Therefore, the presence of melts (metallic, carbonate or silicate) and/or fluids (COH) associated with relevant oxygen fugacity conditions is required to precipitate diamonds in conditions recorded by their inclusions, i.e., closer to the graphite–diamond boundary [33].

Polycrystalline natural diamonds can also be found but are very rare. One of them is the enigmatic carbonado diamond, which is mined for industrial purpose [34]. Briefly, carbonados are black and porous, with 1 micrometer grain size of randomly orientated diamonds and with silicate and metal inclusions as well as a high number of Poly-Aromatic Hydrocarbons (PAH). It is found in Brazil, Central African Republic and Siberia. Origin of carbonados is still being debated and could be either from cold subducting slab [35], from regional metamorphism in Earth’s crust [36] or from seed-heating by fission of radiogenic elements [37]. The last potential origin is formation from meteoritic shocks as polycrystalline diamonds (with nanograins) have been also found near impact craters [30].

Diamond deposits are usually two-fold: primary or magmatic and secondary or alluvial [38,39]. P–T conditions for the formation of diamonds are easily reached in the Earth’s upper mantle. However, there are only few diamond deposits that are in close proximity to ancient cratons, with very stable (no tectonic) old thick continental crust with mantle keel beneath (Figure 2d, [40]).

**Figure 3:**
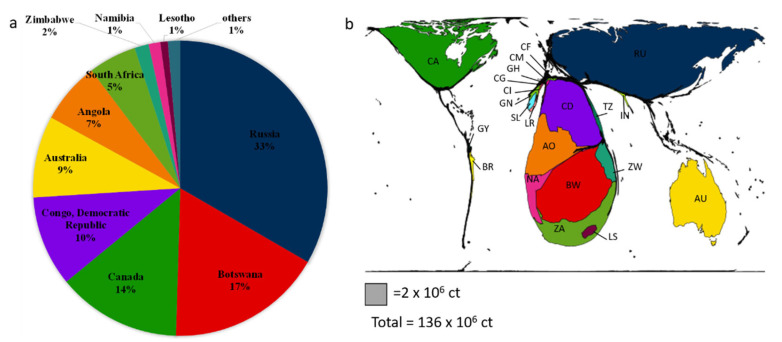
Localization of the main countries producing diamonds through their mines. (**a**) Among the top ten diamond mining countries, seven are from Africa, which reflects the cratonic geological setting. Others represented countries from place 11 to 22 are Sierra Leone, Tanzania, Guinea, Brazil, Liberia, Guyana, Ghana, India, Central African Republic, Ivory Coast, Cameroon and Congo. Once again, Africa is largely represented. Total of diamonds from mine was ~136 Million Carats in 2019. Source: [41]. (**b**) Cartogram representing these 22 biggest natural diamond mining producers, realized thanks to [42] algorithm. RU = Russia, BW = Botswana, CA = Canada, CD: Congo, Republic Democratic, AU = Australia, AO = Angola, ZA = South Africa, ZW = Zimbabwe, NA= Namibia, LS = Lesotho, SL = Sierra Leone, TZ = Tanzania, GN = Guinea, BR = Brazil, LR = Liberia, GY = Guyana, GH = Ghana, IN = India, CF = Central African Republic, CI = Ivory Coast, CM = Cameroon and CG = Congo. It is of note that this map is almost perfectly correlated with the localization of the main craton area.

In most cases, these deposits (primary) are related to kimberlites, a volcanic rock with an ultrabasic composition that is very rich in volatiles (H_2_O and CO_2_), leading to very explosive eruptions. Diamonds are thus upwarded by these brutal eruptions, i.e., by very fast magma ascent, and are concentrated in subsurface pipes and dikes that are exploited today as mines. These deposits are mostly located in Africa, Russia, Canada and Australia, making these regions the biggest natural diamond producers (Figure 3). Other types of primary deposit exist, such as basalt/eclogite in subduction zone, lithospheric mantle and convecting mantle for deep diamonds. Alluvial deposits (secondary) are directly related to primary ones because erosion of these latter would accumulate diamonds in sedimentary traps. Finally, impact-induced diamond formation and ultra-high-pressure crustal diamonds are more anecdotal.

In 2019, about 136 million carats, i.e., 27 tons (1 ct = 0.2 g), was extracted from different mines all over the world [41]. Most of this extraction comes from primary deposits, but the yield in such environment is very low and typically in the order of 1ct for 10 tons of rocks. This is, for example, lower than gold deposits, which are around few grams per ton of rock. Among the 10 biggest producers of natural diamonds, seven countries are from Africa (Figure 3). In addition to having an important environmental impact [43], diamond extraction also has huge societal consequences in these countries, where “conflict diamonds” or “blood diamonds” caused wars, corruption, underdevelopment and death [44,45].

### 3.2. Diamond Classification

Diamonds can be classified according different features that were primarily established for jewelry. Here, we will briefly describe the 4-C rule (carat, clarity, color and cut) and a usual classification based on optical properties (the type classification).

#### 3.2.1. The 4-C Classification

The 4-C rule applies essentially in jewelry. The first C refers to “carat”, the unit that measures the weight of diamonds, with 1 carat equal to 0.2 g. The second C is for “color” and is based on 23 levels (from D to Z), from colorless to light yellow diamond. This can be related to the type classification too (cf. below). The third C is “clarity”, which describes the level of impurity potentially contained in diamonds. Each level is denoted by letters and numbers. Finally, the fourth C characterizes the “cut” of diamonds that will reveal all the brightness and scintillation.

#### 3.2.2. The Type Classification

This classification refers to the optical properties of diamonds and therefore to their impurities as substitutions for their lattice. It has to be noted that this classification suits both natural and synthetic diamonds. Thus, two different types were determined based on the presence or absence of Nitrogen (N) in the lattice, i.e., the most common impurity found in diamonds. For more details about type classification, the reader can refer to Section 6.1 and [46].

**Type I:** In type I diamonds, nitrogen (N) is present in the lattice to a level that can be measured using IR absorption spectroscopy (>500 ppm). Nitrogen in diamond lattice usually gives a yellowish color. This category was also divided into two subgroups according to the arrangement of Nitrogen atoms in the lattice. Hence, Type Ia are characterized by aggregated N impurities in the lattice, either in N-pairs (type IaA) or 4N + V (V for vacancy, type IaB), whereas in type Ib, nitrogen atoms are isolated from one another in the lattice. Most of natural diamonds are type Ia (~98%), and, more specifically, IaA, whereas most of synthetic diamonds using HP–HT techniques are Ib.

**Type II:** In this type of diamond, there is no detectable nitrogen impurity, at least by spectroscopic techniques (<2 ppm). Again, two subgroups were created based on the absence of detectable impurities (Type IIa) or by the presence of isolated Boron (B) in the lattice (Type IIb). Hence, type IIa is considered to be the purest type of diamond; it is also an electrical insulator and is very rare in nature, whereas most synthetic diamonds by CVD techniques are of this type. The boron contained in type IIb gives a blueish color to this diamond, resulting in specific electrical properties that are very rare in nature and in synthetic diamonds.

## 4. HP–HT C-Phase Diagram and the Graphite–Diamond Equilibrium Line

The first HP–HT carbon phase diagram as well as the notion of stability field of graphite and diamonds was provided using thermodynamics [47]. It was then adapted using the principle of metastability [48], which is still under consideration more than a century later.

Still using thermodynamic and specifically calculation of heat of formation [49] and reaction kinetics with temperature [50], the diamond/graphite equilibrium line was estimated. This latter study proposed the conditions for direct transition as well as the effect of using a metal catalyst [50]. The melting temperature of graphite at ambient pressure was also estimated at 4273 °C, a value relatively close to real one.

**Figure 4:**
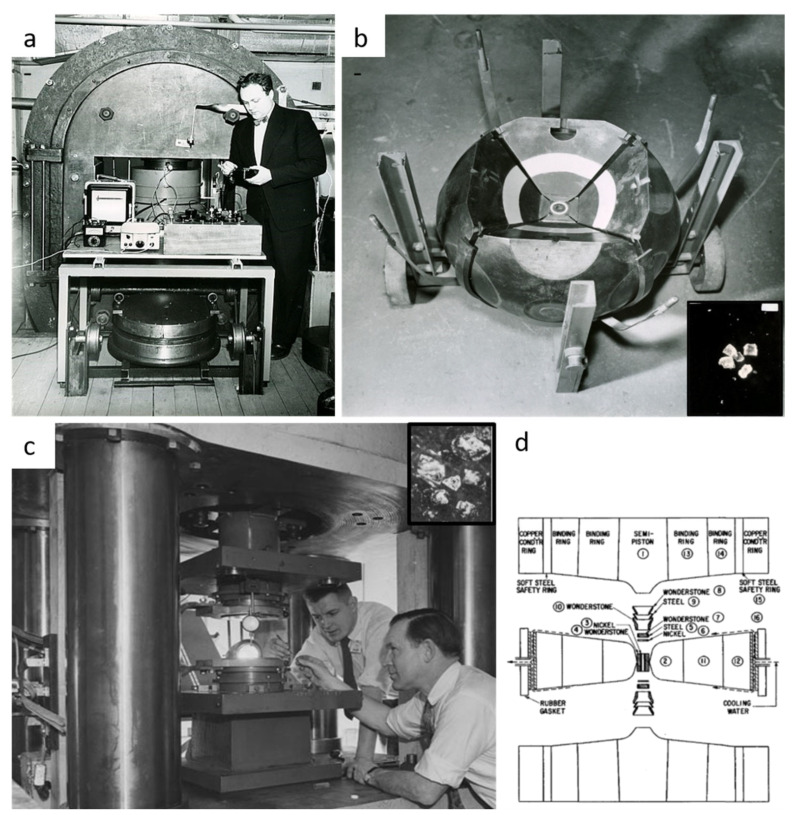
The historical first successful experiments of synthetic diamonds. (**a**,**b**) From ASAE company (Sweden) using the 6-anvils sphere apparatus [53,54]; a-picture from https://commons.wikimedia.org/wiki/File:ASEA_Quintus_press_for_diamonds_1953.jpg (accessed on 1 December 2021)., b-Reproduced from [54], with the permission of AIP Publishing (**c**,**d**) From GE company in 1955 using the famous Hall “belt”-type apparatus [4,55,56]; pictures from https://www.ge.com/news/reports/diamonds-werent-forever-in-the-ge-store-but (accessed on 1 December 2021). Inset are shown the recovered diamond after experiments with a typical size of few hundred microns. These few studies mark the starting point of the development of diamond synthesis for scientific and industrial purposes (Section 5) as well as the building of more and more precise HP–HT phase diagram of carbon (Figure 5).

In 1940s and 1950s, the focus was on the Clapeyron slope of the graphite–diamond equilibrium line with very contradictory results [3,51,52]. It was only in the mid-1950s and early 1960s that graphite-to-diamond phase transition was experimentally observed by ASEA AB [53,54] and GE Company [55,56]. The success of these two experiments was possible after huge instrumental development to reach pressure up to 8–10 GPa and temperature of 2000 °C. In this way, two different high-pressure apparatuses were used by the two teams: a 6-anvil spherical and the famous “Hall” belt-type [4] press (Figure 4). The 6-anvil spherical set up was quickly given up due to handling difficulties and high level of anvil mechanical failure during experiments (Figure 4a,b). On the other hand, the “belt” type apparatus is easy to manipulate, with limited risk of failure and higher reproducibility, making this press the most used for industrial applications (Figure 4c,d). These experiments were performed using iron catalyst and no seeds, resulting in the formation of diamond grains of few hundred microns (inset Figure 4b,c). The very first direct catalyst-free conversion from graphite to diamond occurred a few years later [56], after an important instrumental development allowing pressure of almost 20 GPa and temperature of nearly 5000 °C to be reached using flash-heating techniques. The triple point was determined to be at 12.5 GPa and 4373 °C, and diamonds were recovered from different experiments even though the yield was lower than 60%.

**Figure 5:**
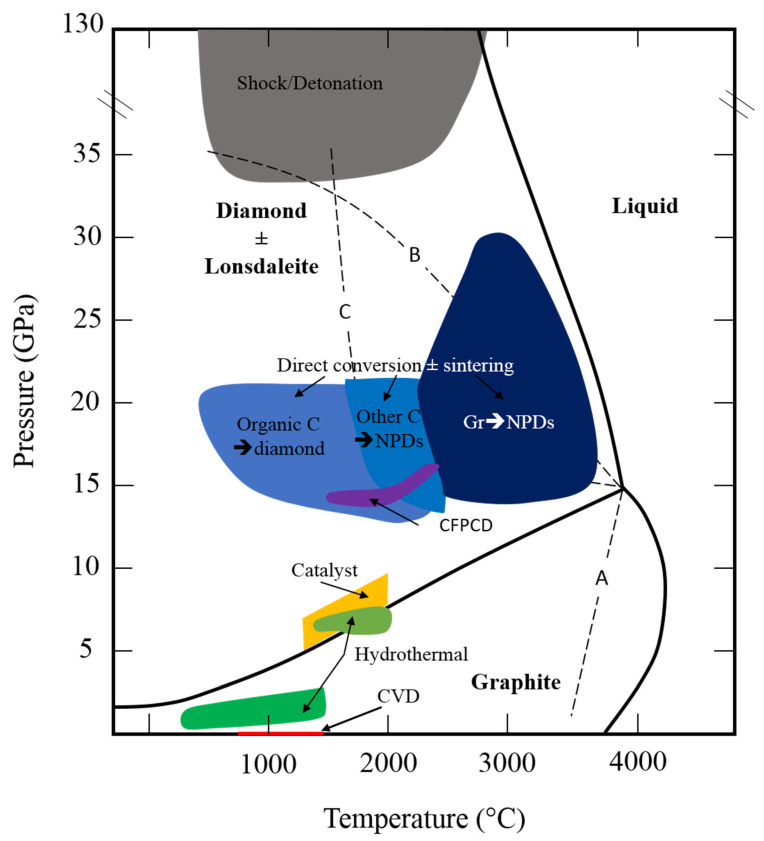
P–T phase diagram of carbon are adapted from [57]. Solid lines are the limits between stability fields. A and B dashed represent the limits between metastability fields, respectively, graphite + metastable diamond and diamond + metastable graphite. C dashed line represents a hypothetical limit between lonsdaleite and diamond stability field. Colored areas show the P–T conditions for the synthesis of diamonds according to the different techniques used.

In the1960s and 1970s, studies of mineral inclusions (HP–HT phases, compositions) trapped in natural diamonds also produced constrains on graphite–diamond equilibrium line (Section 3). Discovery of lonsdaleite in 1967 added complexity to C-phase diagram at high pressure and temperature, and today it is still unclear if this phase is stable or not (Figure 5).

More recently, it was reported that diamonds could be stable up to 2 TPa [58], a pressure at which other stable phases were predicted [59]. This question of diamond stability and/or metastability is thus more than ever under debate and depends on much more parameters than previously thought. Such metastability field is the basis of a lot of different processes for the synthesis and sintering of diamonds under disequilibrium conditions (Section 5).

## 5. On the Way to Catalyst-Free/Binderless Synthetic Diamonds

Since late ninetieth and early twentieth centuries, the original experiments from [2,60,61] tried to synthesis and sinter diamonds in laboratory. A large variety of experimental and analytical set-ups were developed toward this aim that allowed for the refinement of the P–T phase diagram of carbon as well as better understanding of graphite-to-diamond transition mechanisms and their exceptional diamond properties (Figure 5).

The main strategy to fabricate synthetic diamonds was to mimic the natural conditions of formation of diamonds in laboratory, i.e., high-pressure–high-temperature (+/− fluids, static or dynamic/shock) and under low pressure (LP) conditions in H_2_ atmosphere (as an analogue of interstellar environment). The main experimental processes, i.e., static HP–HT, dynamic HP–HT, and LP, to synthesize diamonds, were all developed in the 1950s–1960s, respectively, by [55,62,63]. Static HP–HT and LP (also called CVD) are the only two processes that allow one to sinter diamonds, i.e., to fabricate diamond compact. The other processes, such as shock/detonation, can only produce powders with different purities, sizes and distributions.

More recently, in the 1990–2000s, other experimental strategies, based on the metastability features of diamonds, synthesis and sinter diamonds, were deployed in very unexpected P–T conditions (SPS, hydrothermal or other unconventional set-ups) (see below). Despite the notable originality of these experiments, only diamond powders were produced with highly variable yields and grain sizes (see below).

**Figure 6:**
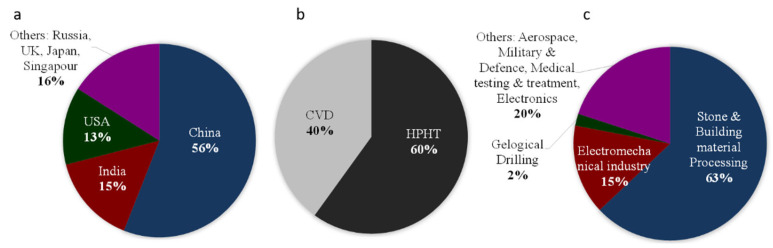
Synthetic diamond production in 2019 represents ~18 billion carats [64,65]. (**a**) By country, China accounts for more than the half of the worldwide production. (**b**) By technique, similar amount is produced using HPHT and CVD. It is of note that China represents 98% of the HPHT synthesis of diamonds. (**c**) By application, from usual tools (e.g., grit, abrasives and drilling) to new technology uses (e.g., in electronics, medicine). Note that synthetic diamond production for jewelry is negligible.

Nowadays, production of synthetic diamonds (also called man-made or lab-grown diamond) is far higher than that of natural diamonds, with worldwide production ~18 billion of carats, i.e., 3600 tons, in 2019, essentially for industrial uses [64]. China largely dominates the world market of synthetic diamonds, with more than 50% of the global production, followed by India and USA (Figure 6a). HP–HT and CVD (LP) are the major techniques used. HP–HT techniques are the leading techniques in synthetic diamonds production. However, CVD process has become increasingly popular following the recent developments to fabricate large pure diamonds [65]. Natural diamonds are essentially used as gems in jewelry (99%), and only 1% are used in industrial applications, whilst synthetic diamonds are widely used for industrial applications and are also employed in various applications [66] (Figure 6c). Among them, about 60% are dedicated to stone and building materials processing, 15% for electromechanical industry and 2% for geological drilling. The last 20% are dedicated to more restrictive applications that are very different, such as military and defense, medical testing, in pharmacy, cosmetic, electronics, high-pressure science, optics and electronics.

In this section, the different experimental techniques for the sintering of binderless diamonds are described, based on a pressure scale, from low to ultra-high pressure. For each of these techniques, starting materials and P–T conditions are described as well mechanisms of formation of diamonds. The yield is also very important and is discussed along with the capabilities of sintering. Special focus is made on HP–HT processes (Section 5.3), techniques that are the most promising for the sintering of big pieces of binderless diamonds.

Indeed, the role of high pressure in synthesis and sintering processes is fundamental [67,68,69]. Concerning synthesis, pressure can stabilize phases at lower temperature or simply phases that do no not exist at atmospheric pressure. Typically, in Earth sciences, it concerns all phases present in deep interiors of planets. In materials sciences, high pressure (±high temperature) is used to synthesize and recover new phases with specific mechanical, optical, electrical or thermal properties that can have many applications.

In terms of sintering, it is known that application of an external pressure on a powder enhances its densification. Indeed, pressure allows for the better packing of grains that can deform and slide to fill free space. Moreover, pores are easily closed by the pressure and therefore the influence of pores’ surface tension becomes negligible. Finally, by applying pressure, other sintering parameters such as temperature and time can be decreased in order to avoid grain growth and obtain objects with better properties.

### 5.1. Low Pressure Processes: CVD Diamonds

CVD technique is, with HP–HT, the only technique that transforms a gaseous carbonaceous compound (CH_4_) to diamond and that leads to a dense body with millimeter thickness. Important developments have been made in the last 30 years to obtain large crystals in terms of surface and thickness (up to few 3–4 mm), with high purity (higher than HP–HT). However, sizes of CVD diamonds remain clearly smaller than those of HP–HT diamonds, either with binder or not. Hence, commercialization of CVD diamonds now represents a large market of synthetic diamonds (Figure 6b).

Briefly, CVD process is a low-pressure–medium-temperature (LP–MT) process that involves ionized gases to allow deposition on pre-existing seeds. Monocrystalline and polycrystalline diamonds can be made with this technique based on the substrate preparation. The first synthesis of CVD diamonds was made by William Eversole at Union carbide in early 1950s using hydrocarbons and carbon monoxide, but the project was given up [70]. The modern origin of CVD techniques was in the early 1960s in three independent labs in USA, USSR and Japan, respectively [63,71]. With these studies, the major role of hydrogen was discovered for synthesis of diamonds by CVD. These studies led to understanding that hydrogen maintains unreconstructed diamond surface structure, i.e., makes possible deposition of new carbon atom (from CH_4_ in most cases) and growth of diamonds crystals at 40 Pa and 1050 °C [62]. During next few decades, it was only possible to grow very thin diamond layers. In the 1980s–1990s, growth rate steeply increased thanks to new technological development of wide range variety of plasma and electric discharges. Thus, four techniques for growing diamonds at low pressure exist [72,73]. All these techniques have their own advantages and potential applications.

***Hot Filament or Foil CVD (HFCVD***). In this case, a metallic filament (W, Ta, Mo or Re, for example) is used to dissociate gases and form radicals suitable for diamond growth. With this technique, the growth rate is typically very low (0.1 to 1 μm/h) but may be increased by the increase of gas flow (as for all techniques).

***Chemical Activation CVD (CACVD).*** Using a combustion flame that permits an exothermic chemical conversion of gases. Here, the growth rate is much higher than in the case of HFCVD and is from 10 to 100 μm/h.

***Electromagnetic Induced CVD***. There are three types of wavelengths used: radiofrequency (RF), microwave (MW) and laser-induced plasma (LIP). A very wide range of growth rates was observed with this technique, from 0.01 to 100 μm/h.

***Electrical induced CVD***. This technique is based on direct-current (DC) plasma discharge between two electrodes. This technique shows the most efficient rate, with most of values between 10 and 1000 μm/h.

Until now, CVD diamonds were the purest diamonds that could be synthesized. Indeed, contrary to HP–HT diamonds, CVD diamonds are type II, i.e., with the absence of nitrogen (see Section 3.2.2). With this aim of purity, recent development has been made to increase the growth rate and control the abundance and nature of chemical and physical defects [74]. Finally, by coupling high growth rate and high purity, fields of application have become important for CVD diamonds, especially for new technologies (e.g., thermal bridges, quantum, and optic windows) rather than in building or cutting tools, which are more dedicated to HPHT diamonds.

### 5.2. Medium Pressure Processes: SPS and Hydrothermal Diamonds

#### 5.2.1. SPS Diamonds

Spark Plasma Sintering (SPS) is a technique involving a pulsed direct current that passes through a powder inserted in a graphite mold (P_max_ = 100 MPa, very high temperature), more rarely in tungsten carbide (P_max_ = 1 GPa, T_max_ = 500 °C). There is a huge literature on this technique to understand densification mechanisms on conducting and insulating samples [75,76]. Briefly, the sample is heated by Joule effect and the current density in the sample produces a “plasma” between particles. This promotes the formation of neck and grain boundaries between them and densifies the powder [77]. This is valid for conducting and insulating material. Therefore, using SPS sintering conditions (temperature, time) to obtain a fully dense body is drastically less effective than the classic hot-pressing techniques [75,76].

With the development and democratization of the Spark Plasma Sintering (SPS) technique at the beginning of 21st century, few studies from one lab have synthesized/sintered diamond at relatively low-pressure–high temperature [78,79,80,81,82,83,84,85].

In their studies, no catalyst or diamond seed was used and different precursors were tested for both the synthesis and sintering of diamonds. It was observed that carbon nanotubes (MWCNTs) and C60 transform partially to diamond at very unusual conditions, i.e., at pressure around 50–80 MPa and temperature above 1150 °C in the vacuum SPS chamber. Using MWCNTs with different purity degrees leads to formation of very fine-grained diamonds, i.e., <10 μm but with unclear evidences from Raman and/or XRD analysis [78,79,80]. Transition from C60 is more obvious, with a yield of 30%, and is thought to be direct, without any intermediate product, and due to some sp3 hybrids present in the C60 [82]. Once all the sp3 bonds of the C60 are converted to diamonds, reaction stops and no more formation and/or growth of diamonds is observed, limiting this process. This typically leads to diamond size of 250 microns in minor content, with the rest of C60 being transformed into graphite, the main element. In any case, no sintering of diamonds particles is observed, and the diamonds are present only as isolated grains. Sintering and stability of nanodiamonds prepared from detonation was also tested using SPS at 60 MPa and up to 1400 °C [84]. It was shown that nanodiamonds remain stable up to 950 °C, but the recovered sample was a loose powder. At higher temperature, nanodiamonds start to destabilize to carbon onion and are fully transformed at 1400 °C. Once destabilized, graphite sintering occurs. This highlights the real difficulty of both stabilizing and sintering diamonds in metastable conditions. More pressure is probably the first parameter to consider.

Either using MWCNT, C60 or nanodiamonds, classic SPS method seems difficult to transfer at the industrial scale. Indeed, the starting materials are expensive, and even though sample size could be large in SPS chamber (>cm^2^) and P–T conditions are very low, transformation is very limited and has not been improved, even using Fe-Ni catalyst [81,85]. Finally, there is a need to isolate diamonds from graphite using strong chemical reagents.

In conclusion, by combining SPS and carbon sources that have already some sp3 bonding, such as C60, a transformation into diamonds particles is possible at unexpected P–T conditions (P ≤ 100 MPa, T~1200–1300 °C), i.e., at very disequilibrium conditions. However, no conversion from sp2 bonds is observed in such conditions. In this context, only isolated nano- to micro- crystals are synthesized without any sintering evidence. A point of interest would be to use other carbon sources as starting materials with a much greater number of pre-existing sp3 bonds such as diamondoïds (fourth bullet of Section 5.3.3). This would probably increase the yield of diamond formation and start the sintering between grains.

#### 5.2.2. Hydrothermal Diamonds

Synthesis of diamonds using hydrothermal techniques was motivated since it was shown that C-H-O fluid could play an important role in the formation of natural diamonds [33,86]. This field of research was of significant interest in late 1990s and early 2000s in order to constrain mechanisms of formation of diamonds in such environments.

From a general point of view, two different techniques were used: a classic hydrothermal treatment at low-medium pressure (LP, MP) and temperatures (LT, MT) (P < 1 GPa, T < 800 °C) and high-pressure–high-temperature conditions (P > 5 GPa and T > 1200 °C) (Figure 3).

Concerning usual hydrothermal conditions for diamond syntheses, three different starting materials can be distinguished, as follows.

**Figure 7:**
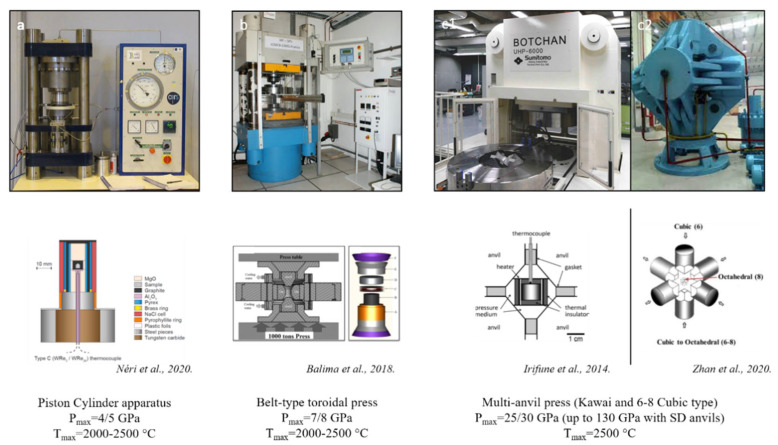
High-pressure devices commonly used for diamond synthesis under high-pressure–high-temperature (HPHT) conditions. From left to right, the maximum pressure achievable increases from 4–5 GPa in Piston cylinder (**a**) to 25–30 GPa in conventional multi-anvil press (**c1**,**c2**). In between (**b**), “belt”-type toroidal press is the most common apparatus used by industrial manufacturer. (**a**) Personal picture, assembly adapted from [100]; (**b**) personal picture, assembly adapted from [101]; (**c1**) picture from http://www.grc.ehime-u.ac.jp/legacy/news79.html (accessed on 1 December 2021), assembly adapted from [102]; and (**c2**) adapted from [103]. (**a**,**b**) are suitable for catalyst-diamond synthesis, whereas (**c1**,**c2**) have been developed more recently for the synthesis and sintering of large volume of binderless diamonds. (See Section 5 for more details).

The first one consists of mixing diamond seeds with different liquids such as 10 M NaOH, C-H-O rich, water, 1,1,1-tichlorethane, hexachlorocyclohexane or dichloromethane. These experiments were mostly performed in piston cylinder (1–2 GPa) (Figure 7a) or Roy-Tuttle autoclave (few hundred MPa) at temperatures up to 1450 °C (mostly to 300 °C) and for duration between 1 and 21 days, mostly 3 days [87,88,89,90,91,92,93,94]. In all these studies, it is unclear if diamonds grow on seeds or not.

The second type of precursor is to use SiC and water, typically under pressure of 100–500 MPa, and temperature between 300 and 800 °C, in a Tuttle-Roy autoclave [95,96,97,98]. In any case, hydrolysis of SiC produced new phases that may be amorphous carbon, graphite and diamonds. It is worth noting too that the diamond yield is very low and that a combination of different analytics was necessary to determine its presence (XRD, RAMAN, SEM and TEM), mostly under nanograins form.

A more anecdotal synthesis of diamonds was to mix in an autoclave at 200 °C for 12 h EDTA, hydrochloric acid (pH = 3) and deionized water [99]. After centrifugation, an almost amorphous phase was recovered, corresponding to approximately 5-nm-sized diamonds.

In all these studies, even though diamond formation was difficult to assess, a range of composition in the C-H-O system was determined for diamond formation. This range of composition was extended under oxidizing conditions [94].

In HP–HT hydrothermal synthesis, graphite was used as precursor and mixed with various fluid generated substances such as water, silver oxalate, oxalic acid, and anthracene [104,105,106,107,108,109]. Similar pressure and temperature conditions have been used in all studies, i.e., P = 5.7–7.7 GPa and T = 1200–2000 °C, and experiments were performed either in “belt”-type or split-sphere multi-anvil apparatus (Figure 4 and Figure 7b,c1,c2). In all these studies, diamond crystals were obtained. Further, diamond formation depends on usual thermodynamic conditions (P&T), on fluid compositions and on duration. Briefly, it was observed that under 5.7 GPa and 1150–1420 °C and under 7.7 GPa and 1400–1600 °C, graphite and diamonds coexist. Moreover, diamond growth rate was almost exponentially temperature-dependent, being hundreds of hours to few minutes between 1150 and 2000 °C [107,108,109]. Diamond crystallization depends also on fluid composition. Indeed, the crystallization rate of diamond decreases according to the following fluid composition: (K_2_CO_3_(Na_2_CO_3_)–H_2_O–CO_2_–C) > (CO_2_–C) ≈ (H_2_O–CO_2_–C) ≈ (H_2_O–C) ≈ (CaMg(CO_3_)_2_–H_2_O–CO_2_–C) > (CH_4_–H_2_O–C_CH4–H_2_–C) [108]. This wide-range fluid composition was also responsible of colored diamonds (colorless, yellow, brown and gray). Finally, yield of diamond crystallization was reported to also be time-dependent with a strong nonlinear dependence [104,105,106]. This suggests that diamond crystallization is due to graphite dissolution to saturation and fast precipitation as diamonds. These studies help one to better understand fluid-assisted natural diamond formation.

### 5.3. High-Pressure Processes: Towards Large Binderless Diamond Objects

#### 5.3.1. Origin in the 1960s

As mentioned above (Section 4), in the mid-1950s early 1960s, two labs succeeded to synthesize diamond from direct conversion of graphite at high pressure and high temperature [53,55]. Later, two studies provided more complete sets of experiments [56,110]. In both studies, graphite was used as a starting material and submitted to pressure between 12 and 15 GPa and temperature up to 3500 °C, by direct heating through the sample by electric current, for very short times (μs) (Figure 5). Recovered samples showed partial transformation to diamond with a yield up to 60%. According to heating method, diamonds were either nano- (20–50 nm) or micro-polycrystalline (10–20 μm). It is to be noted that [56] tried to synthesize diamonds with amorphous carbon, leading to the formation of gray-white polycrystalline diamond, most likely with a higher yield and purity.

This research area was quickly given-up since the role of metal catalyst (Ni based) and the drastic decrease of P–T conditions to synthesize diamonds were discovered contemporaneously [56].

#### 5.3.2. A Brief Description of Catalyst Synthesis of Diamonds and PDC

For the last 50 years, diamond industry has been dominated by catalyst diamonds and associated PDC. Even though it is not within the scope of the present review, a brief description of this process is given.

Indeed, after the discovery of [56], two other studies from General Electric Compagny detailed the C-Ni phase diagram at high pressure and high temperature [111,112]. These two studies defined the graphite–diamond equilibrium line that was later refined from 4.7 GPa, 1100 °C to 6.1 GPa and 1620 °C [113] (Figure 5). With such low conditions, industrial transfer was possible with low-cost large-volume apparatuses such as Toroid press and piston cylinder. It is noted that other catalysts were developed and used (Cu, Zn and Ge [114,115]) until recently with more focus on color center (Mg, Ge, [116,117]). Catalyst diamonds are then recovered from the metallic matrix using acid leaching and are used for grinding, polishing, as cutting wheels or as PDC (polycrystalline diamond compact) for drilling machine or anvils in high-pressure research. In the case of PDC fabrication, catalyst diamonds are then sintered using a binder (mostly Co) at 5 GPa and 1500 °C [118]. This technique is ideal to produce big object of few cm^3^. The amount of binder, as well as the grains size of diamonds, is important for mechanical properties and thermal stability [119,120,121] (Section 6). Typically, hardness of PDC is around 70 GPa and thermal stability is 400–500 °C, which are not sufficient when cutting new materials or drilling rocks.

Therefore, new routes to synthesize binderless diamond compact are necessary but technical, and cost difficulties remain regarding a potential industrial transfer, due to the very high-pressure and high-temperature conditions of synthesis and sintering.

#### 5.3.3. Development in the Last Two Decades

**Figure 8:**
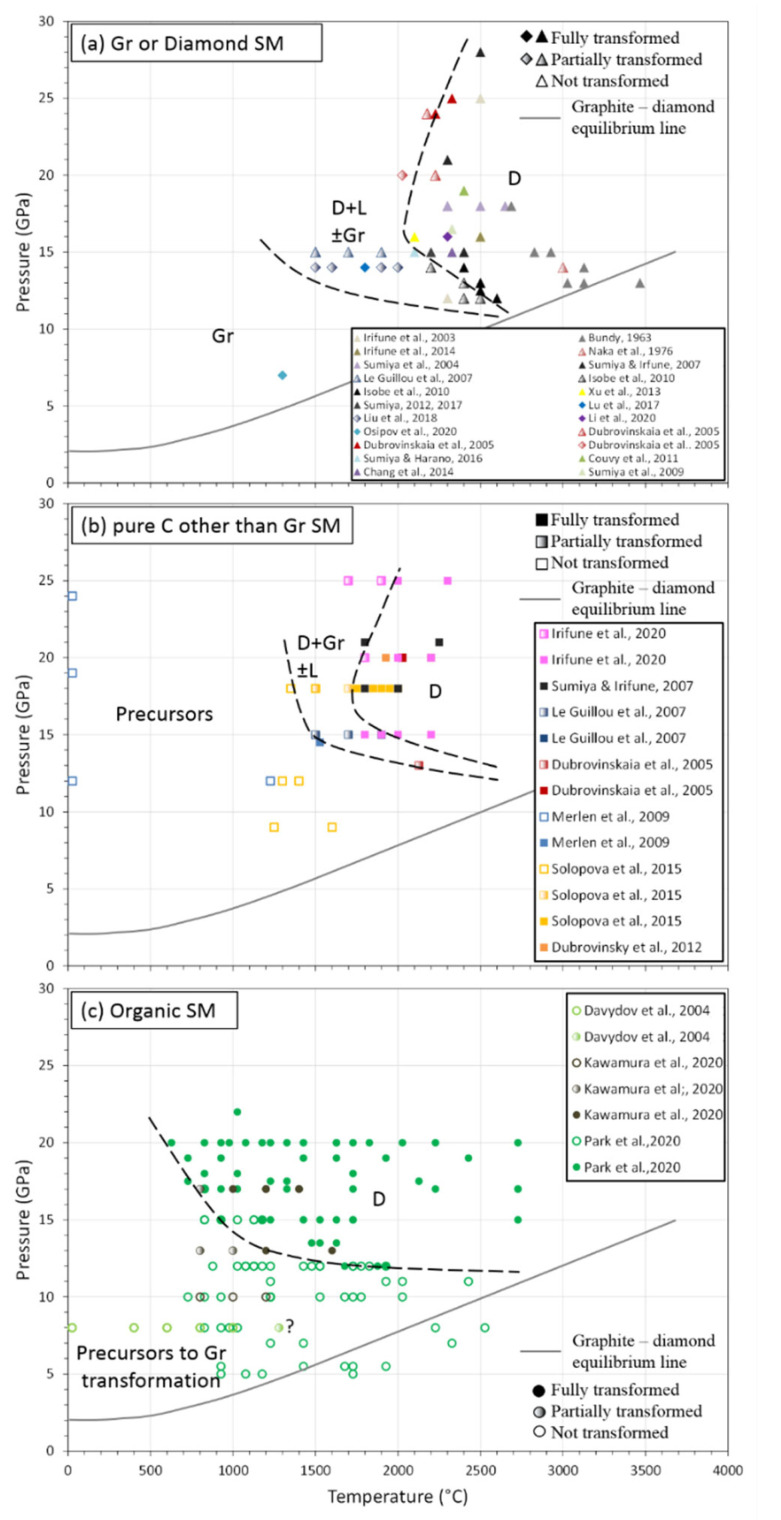
Pressure–Temperature diagram and data compilation of direct transformation of carbon compounds to diamonds at high pressure and high temperature. SM is for starting materials, C for carbon, Gr for graphite, L for lonsdaleite and D for diamond. Filled symbols are for total conversion to diamonds; open symbols are for no transformation; and color gradient is for incomplete, i.e., with SM remaining or for the presence of L in the sample. Triangle and diamonds are for Gr and D SM (**a**), square for other than Gr or D pure carbon SM (**b**), and circles are for organic compounds as SM (**c**). Grey solid line is for the graphite–diamond equilibrium as defined in Figure 5. Black dashed lines are guide for the eyes to note the transition to diamond. It is of note that this transition is favored with organic compounds (**c**) compared to other pure C form (**b**) and to graphite precursor (**a**). Additionally, lonsdaleite appears with pure carbon precursors and seems to be more present with higher crystallinity graphite (see text for more details). References: (**a**) [5], Irifune et al., 2003; [56], Bundy, 1963; [102], Irifune et al., 2014; [110], Naka et al., 1976; [122], Sumiya et al., 2004; [123], Sumiya & Irifune, 2007; [124], Sumiya & Irifune, 2008; [125], Le Guillou et al., 2007; [126], Isobe et al., 2010; [127], Xu et al., 2013; [128], Sumiya, 2012; [129], Sumiya, 2017; [130], Sumiya& Harano, 2016; [131], Couvy et al., 2011; [132], Chang et al., 2014; [133], Sumiya et al., 2009. (b) [134], Lu et al., 2017; [135], Liu et al., 2018; [136], Li et al., 2020; [137], Osipov et al., 2020; [138,139], Dubrovinskaia et al., 2005a,b; [140], Irifune et al., 2020; [141], Merlen et al., 2009; [142], Solopova et al., 2015; [143], Dubrovinsky et al., 2012. (c) [144], Davydov et al., 2004; [145], Kawamura et al., 2020; [146], Park et al., 2020.

In the last two decades, a lot of technical developments have been achieved in order to reach higher pressure and temperature conditions. Thus, binderless diamonds synthesis and sintering was studied a lot in order to fabricate diamonds from different precursors with the aim to obtain large samples with exceptional properties (Figure 8, Table 2).

- Binderless Diamond from Graphite: NPDs (Nano-Polycrystalline Diamonds)

Graphite is the obvious precursor to think about when diamond synthesis and sintering want to be performed.

The first report of binderless diamond compact synthesis and sintering from the direct conversion of graphite is relatively recent [5]. These compacts were fabricated using a multi-anvil apparatus to reach pressure up to 25 GPa and very high temperature of 2500 °C (Table 2, Figure 5, Figure 7 and Figure 8a). The ingenious idea was to use directly dense graphite rods as starting materials that allow for pre-shaping of the resulting diamond compact and avoid cold compression texture/delamination. It is of note that all the following studies have kept this idea (Table 2). A transparent diamond compact was recovered, with limited dimensions of 0.1 mm diameter and 0.3 mm thickness, composed of nano-grains (NPD for nano-polycrsytalline diamonds). These compacts showed exceptional mechanical, optical and thermal properties. Therefore, a wide range of applications could be considered (see below). This first study marks the starting of an important collaboration in Japan between Geodynamics Research Center (GRC) represented by Pr. T. Irifune, and Sumitomo Electric Industry (SEI) represented by Dr. H. Sumiya, head of the Advanced Materials Laboratory [147]. Indeed, important technical developments were made and numerous scientific studies were published in order to understand the mechanisms of this direct conversion sintering. With these new technical developments, bigger dimensions were obtained, and today, it is possible to recover samples of ~1 cm^3^. It is of note that, still today, synthesis, sintering and characterization of NPD from graphite are almost exclusively performed by these two Japanese labs.

NPDs microstructures sintered between 15–18 GPa and 2300–2700 °C were studied (Table 2, Figure 8a), mostly by TEM and associated techniques, to understand mechanisms of the direct conversion sintering diamond body [122]. This study revealed the almost systematic presence of lonsdaleite in the final product. However, its abundance depended on sintering temperature essentially and was detected either with XRD or by electron diffraction using TEM, respectively, at low or high temperatures. Nevertheless, NPDs had a mixed texture of homogenous fine or lamellar structure, the former being composed of randomly orientated 10 nm grains, the latter being inherited from originally deformed graphite. This lamellar structure was most likely responsible for NPD’s hardness compared to sample without this structure (Section 6, see below, [123,124]). This lamellar structure disappears at very high temperatures when grain growth becomes efficient and new grain boundaries are formed. This dual texture was explained by two different mechanisms of transformation, respectively, by diffusion and by martensitic process. To go further with understanding of transition mechanisms, the authors of [125] characterized transformation from graphite and different precursors submitted at 15 GPa and between 1500 and 1900 °C (Table 2, Figure 8a and third bullet of this section). It was shown that the presence of lonsdaleite is highly dependent on the ordered structure, i.e., crystallinity of the precursors, i.e., the most ordered the structure, the more lonsdaleite was present. In all products, nano-diamonds were formed, but the degree of transparency depends on P–T conditions. Transformation mechanisms were determined by using different type of starting graphite (more or less orientated grains in the rod). These mechanisms mostly act simultaneously and are diffuse and related to defects and restacking.

Many different experimental conditions were tested to determine the lowest P–T conditions of this direct conversion sintering from graphite. It was shown that transition could occur at relatively low pressure of 12 GPa, but it needs temperature as high as 2600 °C and that therefore higher pressure means lower temperature (Table 2, Figure 8a, [126]). Moreover, in most of these “low”-pressure range experiments, lonsdaleite was found, except at very high temperatures (Table 2, Figure 8a). At high temperatures, grain growth was also present and the lamellar structure disappeared, making lower hardness compacts as suggested previously [123,124]. Another study tried to decrease P–T conditions of this direct conversion sintering from graphite to diamond by comparing NPDs from crystalline graphite and milled graphite after sintering at 16 GPa and up to 2500 °C (Table 2, Figure 8a, [127]). From crystalline graphite, lonsdaleite was always present, whereas it was absent using milled graphite [127], confirming that the structure ordering is of major importance, as also underlined by other precursors (third bullet of this section). A thermodynamic and kinetic process were considered to explain this difference with different transition states according to the precursors, and therefore low overpressure was necessary to start the transition.

The best conditions for this direct conversion sintering from graphite to give transparent NPDs with very high mechanical properties were then defined (Table 2, Figure 8a) and are P > 15 GPa and 2100 < T < 2400 °C [128,129]. Lower temperatures produce too much lonsdaleite and insufficient densification, and higher temperatures promote grain growth and disappearance of lamellae texture. Even though mechanisms of transformation can be slightly different as already exposed, the main feature is the presence of lamellar texture in final samples, a structure that is only observable with graphite precursors, even if lonsdaleite is present.

- Sintering from Diamonds Precursors

Twenty years ago, Voronov, associated with the DMI Company, proposed to sinter diamond compact without any binder using only diamond powders [148]. The main goal was to use binderless polycrystalline diamonds (BPCD) for drilling applications and thus avoid thermal problem of conventional PCD (presence of Co-Binder). Accordingly, a 300-ton toroid press was used and an important development of the anvil materials (nano-grained WC/Co was made to support pressure up to 15 GPa and temperature up to 2000 °C. However, with the aim of an industrial transfer, all experiments were performed between 7–9 GPa and 1000–2000 °C and for duration between 1 and 100 seconds. An important work on precursors was made [148]. Different types of diamond (natural, HP–HT and detonation) with different grains size (1 nm to 50 μm) and purity (0.1 to 20 wt% of contamination, i.e., in most cases Fe, Ni, Cu, Mn, Si O, N and H) were tested, as well as the mixing of different grain-size populations. Thus, pellets of 100 mm^3^ (6 mm diameter and 3 mm high) were synthesized and characterized using XRD, SEM and EDS. Even though dense bodies were recovered (ρ = 3.1–3.5 g/cm^3^), analysis not only showed diamonds in the aggregates. Indeed, in XRD pattern, an important background to the lower angle was visible, suggesting the presence of an amorphous phase that could be a carbon phase. This was confirmed by SEM and EDS analysis, which revealed the presence of ashes in the aggregates with composition rich in C and metal elements. Finally, no Raman spectra were shown for the presence and/or absence of graphite, which is an unavoidable analysis to characterize diamonds. These first binderless PCDs had similar properties than conventional PCD, e.g., a thermal stability up to 1200 °C in Ar atmosphere, a thermal conductivity to 600 W/m.K, a Vickers Hardness of 75 GPa, a strength of 3.8–4.2 GPa and a limited abrasive wear, making them not much better.

This protocol was retried only by two different teams almost twenty years later, which were distinct due to the choice in their starting powders and conditions of sintering.

Recently, a lab from Sichuan University, China put a lot of effort into directly sintering micrometer-sized diamond powders into dense bodies with several mm^3^ volume. These bodies are referred as MPD (micro polycrystalline diamond) or CFPCD (Catalyst-Free PolyCrystalline Diamonds), meaning binder-free. All these compacts have been synthesized in hinge-type cubic press (Figure 7c).

The first study from this lab used high-purity commercial diamond powder with initial grain size of 0.5 mm, which was substantially re-purified. Sintering at 14 GPa and up to 2000 °C has shown that temperature is very critical regarding the diamonds back transformation to graphite, even for sintering time of 1 min (Table 2, Figure 8a, [134,149]). With this patent [149] and first study [134], mm^3^ diamond pieces have been achieved with very high hardness and grain size of 170 nm, showing the milling of diamond powders during compression.

More recently, further developments were made to better control and identify the sintering mechanisms. Using previously purified 8–12 μm grain size of diamonds that were pre-compacted into a pellet with a relative density of 75%, it was shown that fully dense diamonds compacts could be sintered at 14 GPa and 1600 °C [135]. By covering a wide range of temperatures, it was shown that graphite appears at 1500 °C and above (Table 2, Figure 8a), especially at triple junction. These areas are thought to be low-pressure zone between high-stress diamonds grains, allowing a back-transformation to graphite. However, temperature had to be higher to avoid brittle behavior, even at high pressure, and to allow plastic deformation of diamonds grains and therefore creation of grain boundaries. Region around these newly formed grain boundaries had shown high level of dislocation, twin boundaries, stacking fault and a lot of diamonds lamellae, guaranteeing a high level of sintering. Triple junction zone contained nano-diamonds grains in turbostratic graphite and amorphous carbon matrix. This is probably the reason why these diamond compacts are opaque compared to that sintering by direct transformation of graphite (first bullet of this section). There is therefore a subtle balance in the temperature control to avoid too much graphite in triple junction but to allow the formation of strong grain boundaries.

The two other studies span the P–T range and focus on the P–T path (Table 2); the final size of the bodies; and the mechanical, abrasive and cutting properties of these diamond compacts [103,136]. For instance, samples up to ~500 mm^3^ (10 mm diameter and 6 mm high) were synthesized using similar set-up and at pressure and temperature up to 16 GPa and 2300 °C [136]. The final product was optically dark, with 10 μm grain size and with the presence of micro cracks. Moreover, XRD and Raman spectra appear very clean, suggesting high-purity samples.

Another lab from St. Petersburg, Russia, tried to directly sinter diamond compact from diamonds powders but using nano-diamonds powders of ~25 nm from detonation synthesis, i.e., with TNT/RDX (Section 5.4) as starting materials [137]. The main goal focused on nitrogen-vacancy-nitrogen-center diamonds for biomedical applications. A precise procedure of purification of the starting materials was developed to remove residual metals and amorphous carbon in the polycrystalline nano-diamonds powders. A mixture of this powder (50–70 wt%) and ethanol (30–50 wt%) was then packed in a graphite cylinder (4 mm diameter and 5.5 mm high) and put into a toroid-press (Figure 7b and [150]). Sintering conditions were P = 7 GPa and T = 1300 °C for 10 s, lower than all other methods of binderless diamonds sintering (Table 2, Figure 8a). With this method, no dense bodies were sintered, but sintering of several grains was observed, and with the presence of ethanol, purity was highly enhanced, which was observable in the change in color from grey/black for the precursors to white/transparent after sintering.

- Sintering from Other Pure Carbon Forms

Other sources of pure carbon were used to synthesize diamonds at high pressure-and high temperature. The main goal of these studies was twofold: first, try to decrease P–T conditions for the formation of dense bodies and then provide new constraints on the mechanism of diamond formation and precise the P–T phase diagram (Table 2, Figure 8b). Thus, during the last 15 years, numerous studies have been reported with these precursors.

Two different types of experiments have been performed.

C60 was the first other pure carbon form to be tested for the synthesis and sintering of diamond compact [138]. P–T conditions were similar to those used with graphite precursor [5], i.e., 20 GPa and 2200 °C for 60 min. Therefore, no difference was observed in the final product, i.e., pure diamond. However, the transparent aggregate (1.8 mm diameter and 3 mm high) was composed of diamond nano-rods of 20 nm of diameter and 1 μm length. This first study was immediately followed by a more complete investigation of another pure carbon source [139]. C60, amorphous carbon (aC) was used under various P–T conditions, i.e., 13–20 GPa and 27–2127 °C, for 60 min (Table 2, Figure 8b). Except at ambient temperature where C60 remains stable even at pressure to 20 GPa, all other C60 precursors had transformed to nano-polycrystalline diamonds, and small abundance of 6H polytype that corresponds to another stacking of C-tetrahedra was present. Moreover, it appears that diamonds forming from C60 have a higher thermal stability, up to 1627 °C, i.e., 300 °C higher than that sintered from pure graphite. Starting with aC leads to the formation of diamond and lonsdaleite at 20 GPa and 2027 °C. Mechanisms of transformation from other pure carbon sources were also established [125]. By using QAC (quasi amorphous carbon), CB (carbon black ± heat treated) and two types of graphite (first bullet of this paragraph) as starting materials and submitted to 15 GPa, at 1500–1700–1900 °C and between 15 and 60 min, it was proposed that the degree of ordering in the precursor was of major importance for the resulting products (Table 2, Figure 8b). Globally, for given P–T conditions, disordered precursors react faster, without any graphitization step, achieve higher transformation rate and are more easily transparent. Under these conditions, nucleation is the dominant mechanism, and these precursors mostly evolve according to a diffusion-limited reconstructive mechanism, which originates from the precursor structural defects. Moreover, even though some reactions were incomplete, in no other phase were diamonds or residual precursors found, i.e., there was no lonsdaleite (Figure 8b). Here [123], aC, GC and C60 were submitted to 18–21 GPa, 1800–2250 °C, for 6 to 35 min (Table 2, Figure 8b). All were 100% transformed to nano-polycrystalline diamonds, with grain size 5–200 nm depending on annealing time. However, TEM observations did not show the presence of lamellar as in diamonds obtained from graphite (first bullet of this section, [123]). Finally, a recent study based exclusively on GC constrained grain size evolution of NPD as a function of P–T conditions and discussed related mechanical properties [140]. It was shown that full transformation temperature increases from 1700 to 1900 °C with the increasing of pressure, from 15 to 25 GPa (Table 2, Figure 8b). Moreover, grain size evolution after 20 min of annealing is highly inhibited at 25 GPa compared to 15 GPa. Therefore, to keep the exceptional properties of NPD with grain size less than 10 nm, very high pressure resulted in lowering temperature and thus limited grain growth. Again, with such other pure carbon starting materials, no lonsdaleite was observed (Figure 8b).

One of the lowest transitions to diamond with pure carbon precursors was established using SWCNT at 14.5 GPa and 1527 °C ([141]; Table 2, Figure 8b). In this study, micro-grain size diamonds formed, and an intermediate step of graphitization to highly disordered structure occurred before transition to diamonds. Again, this disordered structure seems to be the source of a lower and direct transition to diamonds.

Other studies, mostly from BGI lab (Bayerisches GeoInstitut), used different precursors to synthesize diamond micro sphere to be used further as double-stage in diamond anvil cell (DAC). These experiments were performed in large volume press in order to mix precursor powders and pressure medium powders of NaCl, MgO or Na_2_CO_3_ [142]. This mixture helped shape the resulting diamond. Only glassy carbon (GC) (20–50-micron size) was used in these experiments. Up to 20 GPa and 2000 °C, GC transformed more or less into pure diamond without any trace of lonsdaleite (Figure 8b), most likely due to the disordered structure of the starting materials, as suggested by others studies [123,125]. The resulting micro-balls of diamonds (10–50 μm) were nano- to micro-polycrystalline and were used in double-stage DAC to reach pressure of 600 GPa [143].

To sum up, diamond transition using other pure carbon precursors occurs at lower P–T conditions than diamonds fabricated from graphite. Moreover, a narrower field of lonsdaleite is observed compared to the one observed with pure graphite as starting material (Figure 8b). These two features (lower P–T conditions and narrow lonsdaleite field) are due to the disordered structure of the precursors that decrease activation energy of nucleation.

- Sintering from Carbonaceous Organic Compounds

Organic compounds transformation were studied a lot, first at high temperature [151] and then at pressure that did not exceed 1 GPa [152], revealing the steps of carbonization and graphitization.

More recently, high-pressure–high-temperature experiments were performed on different types of organic precursors. Here is given a brief overview of three complete studies [144,145,146] that span over a wide range of pressure–temperature conditions (Table 3, Figure 8c).

Different PAH (polycyclic aromatic hydrocarbons), i.e., naphthalene, anthracene, perylene and coronene, were submitted to 8 GPa and up to 1500 °C for 60 s in a toroïd type press [144] (2, Figure 7b and Figure 8c). Special care was taken for the choice of container. Graphite was chosen instead of metal to avoid any catalyst action and to allow produced gas to escape due to the porosity of graphite. XRD, SEM and Raman results did not show that any difference based on the PAH used. In any case, a step of graphitization occurred at 800 °C and diamond transformation started around 1280 °C, which was incomplete transformation. Typically, diamond grains of 5–40 μm were formed. A crystallographic link was observed for the graphite-to-diamond transformation, with diamond facets perpendicular to the graphite platelets. Such conditions of transformation are the lowest known without any catalyst to date.

Experiments in multi-anvil press using stearic acid (C_18_H_36_O_2_) were performed at P = 10–17 GPa and T = 800–1600 °C and for 5 to 180 min [145] (Table 2, Figure 8c). The main goal was to fabricate at lower conditions nano-polycrystalline diamond (NPD) compact with similar quality (grain size, transparency) to those classically formed using graphite (first bullet of this section). In order to obtain dense bodies without any pores, water from stearic acid dehydration has to be removed from the sample using MgCO3 porous powders under the sample that allows percolation. With this set-up, transition to diamond started at 13 GPa and T = 800 °C and was achieved at 1200 °C, clearly lower than NPD from graphite (T > 2000 °C) (Figure 8a). Moreover, no lonsdaleite was observed, which was in agreement with an intermediate, highly disordered, amorphous graphite stage during heating that promoted the formation of diamonds. Thus, activation energy of transition was reduced, even more by the presence of CH_4_ and H_2_O.

Different diamondoids (adamantane, diamantane and triamantane) were tested to be transform into diamonds [146,153,154]. Diamondoids are very small H-terminated carbon cages (few nm) with a lattice structure of diamond. Pioneering works by [153] showed that diamondoids transformed to diamonds at 12.5 GPA and 200 °C, values that were clearly lowered to 8–9 GPa and 1300–1600 °C [154]. Using laser-heating DAC and time-resolved XRD (<19 μs), these phases were submitted to pressure between 5 and 22 GPa and temperatures between 627 and 2727 °C (Table 2, Figure 8c) [146]. No obvious difference was observed for the different precursors, and transition to diamond occurred at 12 GPa and 1727 °C and 20 GPa and 627 °C for the lower limit in pressure and temperature, respectively (Figure 8c). No graphitization step was observed for the mechanism of transition, but further ab initio calculations have suggested that once diamondoids were dehydrogenated, they bond together in the diamond structure. This is therefore a direct sp3-sp3 reconstruction.

It therefore looks quite easily to form diamonds from organic compounds. Indeed, transition is largely lowered compared to graphite or other pure C precursors. Moreover, transition is direct, without any lonsdaleite field, again probably due to the disordered structure of the pressurized starting materials. However, to the best of our knowledge, no data exist on the mechanical, optical and thermal properties of these bodies.

### 5.4. Ultra High Procesess: Shock and Detonation Diamonds

Diamonds produced by shock and detonation were studied in order to understand observed diamond in both impact crater and some meteorites and thus provide constraints on their conditions of formation. The first report of diamond formation by shock from graphite was made by [61] at 30 GPa for 1 μs, with a relatively low recovery, but diamonds particles observed were <10 μm diameter and well identified using XRD. Moreover, due to the very fast shock wave, the mechanism of transition was thought to be diffusionless and most likely due to the compression of c-axis leading to atom rearrangement through the cubic diamond form.

This pioneering study was the starting point of production of diamonds by shock using mainly three different techniques that will be shortly described below. The main applications of these shocked diamonds concern polishing using nanograins [155] and more recently have been devoted to bioapplications [156].

The first technique uses a shock wave that is produced either by an explosion, by a projectile or by a laser. In most cases, precursor was graphite with different grade/crystalinity [157,158,159,160]. Other materials such as fullerene [161], black carbon, amorphous carbon, adamantane [162] and even rock containing graphite [163] were also used. In all these experiments, the yield of dense phases was always very low, i.e., 5–10% maximum. In experiments containing graphite with different crystallinity and at pressure between 20 and 228 GPa (Figure 5), it was shown that lonsdaleite is preferentially formed if graphite is pyrolytic, whereas diamond forms even with more disordered graphite. Moreover, lonsdaleite formation seems to be favored when graphite basal plane is perpendicular to the shock wave, whereas diamonds are formed regardless of graphite crystals orientation [159]. Transition was most likely martensitic as the shock wave was very fast, and a resolution of 10 ns was allowed to follow these transitions [159]. However, lonsdaleite remained unstable as temperature increased in favor of stable cubic diamond (T > 3000 K). With other precursors, only diamonds were formed, with micrometer size (6–10 μm).

Second method involved using the carbon contained in explosives, which was transformed into nano-diamonds in a closed system that generated suitable conditions of formation for diamonds. It has to be noted that there was no lonsdaleite form in these experiments due to the low degree of crystallinity of these explosives. The pioneering experiments by [114] have shown that, according to the explosive (TH50/50 or TNT) used, the yield for diamond recovery varies from <5% to 40–50%. In this study [164], P = 18–19 GPa and T~3700 °C, and the transformation occurred in 0.5 μs (Figure 5). It was also proposed that transformation involved kinetics processes rather than equilibrium ones. In order to control this kind of transformation by explosion, especially concerning grain size and distributions, several technical developments were proposed in the last two decades. For example, inert gas was replaced by water in confinement bag that allowed for a rapid cooling that avoided back transformation to graphite [162]. In that case, the yield reached 60% when using TNT/RDX +/− HMX/TNT. A lot of efforts were also made on the best ratio TNT/XRD as well as on the nanostructure of these explosives in order to produce the smallest particles (down to 3 nm) with a very narrow distribution involving diffusive processes [165,166] that can influence optical properties [167]. Purification procedures were also developed to increase the yield [165]. Other explosives (BTF, C_6_N_6_O_6_) were also tested to produce hydrogen-free nano diamonds [168] in order to access specific properties.

Last but not least, the third method involves sintering diamond (12 mm diameter and 5 mm thick pellets) from diamond powder using shock without any binder [169]. To avoid the problem of cracks, shock residual temperature had to be controlled as well as the starting grain size. The development of cracks increased with the increase of grain size, and relative density reached 90% for bigger grain size.

A final attempt was to produce nanodiamond using laser techniques [170]. This process is similar to a shock but differs by the fact that the high energy laser is focused on the solid target that is placed in a liquid (mainly water). This technique is very common for a lot of metal and ceramics. For nanodiamonds synthesis, the interaction between the laser and the graphite (i.e., the target) vaporized this later. Crystallization occurred in contact with the liquid at HP–HT, synthesizing nanodiamonds with grain size from few to hundreds of nm.

### 5.5. Other Unconventional Expeirmental Set-Ups

This section describes original unconventional techniques in which diamond formation was reported for different conditions of pressure and/or temperature. However, these new findings have to be confirmed since only single study was devoted to a given technique.

Ultrasound cavitation was used to synthesize micro-grain (6–9 µm) diamonds from graphite (100–200 μm) in various organic solvents, with a significant yield up to 10% [171]. Although authors claimed that synthesis was made at 1 bar and 120 °C, real local conditions inside the cavitation chamber are most likely to be thousands of degrees Celsius and several GPa. One of the main advantages of this technique was the fast transformation, around 10 min. The mechanism of transformation in this method was “probabilistic” and based on the number of cavities that collapsed normally onto the graphite basal plane. This kind of “probabilistic” approach makes it unsuitable for an industrial transfer, even though this technique seems to be energetically favorable to synthesize diamond, similar to HP–HT and clearly better than CVD [171]. No other work was reported to demonstrate the reproducibility of this phenomena, inducing certain reservations with regard to the technique.

Diamond nanorods were also fabricated from carbon nanofibers using pulsed laser annealing [172]. Once again, authors claimed that transformation occurred at ambient pressure and temperature, whereas this conversion involves melting of carbon nanofibers under a super undercooled state. Pressure was also most likely high due to repeated laser pulse that controlled the rate of transformation. Complete transformations occurred using this technique, and resulting diamond nanorods can be used as seed for further CVD diamonds synthesis [172]. Additionally, in this case, no other works were reported to demonstrate the reproducibility of this phenomena, inducing certain reservations with regard to its reproducibility, although it was reported to have a significant yield of 100%.

High shear strain, using a rotational anvil apparatus, under ambient temperature and pressure below 1 GPa (0.4–0.7 GPa), allowed for the direct formation of diamond from graphite powder [173]. In this study, lonsdaleite was also observed. In any case, transformation was far from being complete (most likely ~1% yield), diamond crystals formed were smaller than 50 nm and a lot of phases coexisted. Moreover, sample thickness did not exceed 50 μm. Interestingly, the mechanisms of formation at such low pressure and temperature came from the concentration of high stress at the limit of lattice instability, which promoted phase transition.

Decomposition of SiC using chlorine-containing gases at 1 bar and 1000 °C in a quartz sealed tube allowed for the formation of nano- and micro-crystalline diamonds [86]. These diamonds were produced as very thin layer, and the interface of SiC and Carbon had hardness properties of 50 GPa and Young modulus of 800 GPa. In this set-up, hydrogen was assumed to stabilize the conversion of SiC to diamond.

## 6. Diamonds Properties and Uses

### 6.1. General Properties

Diamond structure is the basis of all of its properties and therefore of its associated uses. Indeed, all is in its tetrahedral covalent bonding forming sp3 hybridization as well as its compacted *fcc* lattice. The level of impurities and defects in the lattice is also of great importance for specific optical or mechanical applications. These features may be altered in natural diamonds or during the synthesis and sintering processes (Section 3 and Section 5).

Diamond has exceptional mechanical properties (Table 3) such as very high hardness, elastic properties, low friction coefficient and low wettability. Specifically, a body of synthetic diamonds called “compact” without binder has higher hardness than natural single crystal due to the presence of grain boundary and especially if the grains are very small (Hall-Petch relation) (Section 6.2, Table 3). Moreover, the recent development of binderless diamond compact with nano-grains (nano-polycrystalline diamonds NPD) shows exceptional wear resistance, transverse rupture strength (TRS) compared to conventional PCD (containing Co-binder), and temperature of 1000–1200 °C (compared to 400 °C for PCD) [130] (Section 6.2, Table 3).

Diamonds are also known to be good electrical insulators [174] (Table 3). Exceptions are for type IIb diamonds (boron impurities between 5 and 10 ppm) [175], either natural or synthetic, that are semiconductors, with a transition to superconductivity around 4 K [176]. Despite their electrical insulator feature for most of diamonds, they also have high thermal conductivity that can be modified in synthetic diamonds (Table 3).

Diamond also possesses the highest thermal conductivity, even higher than copper [177]. This is even truer for synthetic diamond enriched in ^12^C compared to natural ones [178,179] (Table 3). Indeed, this property depends essentially on defects that are present in the crystal (e.g., minor phase, dislocation, lattice substitution, and vacancy) and that can be controlled in synthetic diamonds, especially in CVD ones. Coupled with its high electrical resistivity, this makes diamonds the best materials for heat sink applications in high-power electronic devices.

Optically [180], diamond is transparent from UV to far IR, with an absorption band between 2.5 and 6 μm. It is also transparent to X-ray and hyperfrequence domain. Thus, it is highly used for optical windows and polarizers. For such uses, the compact may not contain any physical defects such as porosity.

By combining its high resistivity, high thermal conductivity and electric properties, diamonds can be used for specific devices such as sensors and detectors [181].

### 6.2. Specific Properties of Binderless Diamonds

Mechanical, optical and thermal properties of these NPDs have been extensively studied over the last decade, and a Special Issue on high-pressure research has been dedicated recently concerning scientific application (HPR, 2020) (Table 3). When possible, comparison with MPD (binderless diamonds sintered from diamond powder) and classic PCD are provided.

- Mechanical properties

When talking about mechanical properties and especially hardness measurements of materials, one has to be aware that determined values depend a lot on the method used, i.e., Vicker’s [182] vs. Knoop’s [183] vs. Nano’s Hardness [184] vs. ultrasonic spectroscopy [185], as they measure these properties at various spatial scales [186].

A lot of studies have focused on elastic properties and hardness of NPDs. Each time, these studies have brought new data on sintering conditions and also constrain mechanical properties on these NPDs.

Knoop hardness (H_k_) was one of the first measured properties on the NPDs [124,125]. Its mean value was determined to be H_k_~120–130 GPa, which was higher than single crystal and higher than other NPDs sintered from other pure C precursors (H_k_~70–120 GPa) (Table 3) (third bullet of the Section 5.3.3). Such high hardness was directly related to lamellar microstructure observed only in samples fabricated from graphite. Moreover, such high hardness was weakly temperature-dependent compared to single crystal, in which, according to orientation, a stark decrease in H_k_ was observed around 200–300 °C [129]. It is of note here that MPD (second bullet of the Section 5.3.3) has hardness at least the same as diamond single crystal (SC) (H_k_ ≥ 120 GPa) as indenters have been broken very often [103]. Moreover, they have similar hardness (H_k_ ≥ 120 GPa) to the more studied NPDs (Table 3) [129].

Ref. [131] measured young modulus (E) and nano-hardness (H_N_) of previously sintered NPDs using nano-indentation technique [131]. NPDs fabricated at 19 GPa and 2400 °C for 30 sec (Table 3, Figure 8a) were very dense and transparent and led to E = 707 GPa and H_N_ = 62 GPa, which was harder than other commercially available polycrystalline diamonds. Other elastic properties (bulk modulus: K_s_, shear modulus: G, and Young’s modulus: E) were determined by using ultrasonic measurements (P and S-wave velocity: V_p_, V_s_) [132] on the three NPDs sintered at 15 GPa, 2327 °C and for 20 min (Table 3, Figure 8a). This bulk method of characterization gave a relative density ρ > 99.94 % and values of V_p_, V_s_, K_s_, G and E, which were very close to those of single-crystal diamond (Table 3).

Other techniques, non-destructive, can be used to determine elastic properties of diamonds and more specifically of synthetic diamonds. The main advantage of these methods is that they measure properties of bulk samples and only of a small area, as for the hardness measurements. For example, ultrasonic methods (RUS: [187] and pulsed-echo: [188]) were used to measure elastic moduli on both natural and synthetic diamonds. These two studies pointed that crystallographic directions have higher elastic properties than others despite the fact that diamonds have cubic lattice, and that polycrystalline diamonds can exhibit similar elastic properties to single crystal. Concerning NPDs, measurement of NPDs stiffness by Brillouin scattering confirmed higher properties than single-crystal diamond [189]. It is of note that in this study, ab initio calculations show that lonsdaleite could be even stiffer than NPDs, a conclusion that has to be confirmed with experiments.

- Thermal properties

Thermal properties of NPDs are at least as high as single-crystal (SC) diamonds, as revealed by XRD up to 1500 °C–1600 °C [128]. At this temperature and under inert atmosphere, NPDs and SC start to destabilize, whereas binder PDCs start to deteriorate at 400–500 °C with the appearance of cracks, and at 700 °C with the appearance of graphite.

Thermal stability of MPDs was shown to be very high, with a starting transformation to graphite at 1200 °C, at least 200 °C above all other natural or synthetic diamonds (single crystal, NPDs…) and twice the thermal stability of common binder PDC [103].

Transverse rupture strength (TRS or flexural strength) of NPDs were measured at both ambient temperature and up to 1200 °C and compared with other diamond materials such single crystal (SC) or PDCs [128,129,130]. At ambient temperature, TRS of NPDS was about 3 GPa, i.e., 15–20% higher than PDCs, and was twice that of single crystal. With increasing temperature, TRS of PDCs drastically decreased to 0 at 500 °C, whereas in the case of NPDs, stable TRS remained up to 1000 °C and tended to increase above.

- Machining and Cutting properties

Plastic behavior of NPDs compared to conventional PDCs was studied at HP–HT using D-DIA press coupled with X-ray radiography and diffraction [190,191,192]. Deformation of PDCs was highly dependent on the binder content and resulted in strong work hardening. However, strength of PCDs was weaker than that of single crystal due to the presence of binder, which acted as a lubricant. On the other hand, NPDs showed a strength 2–3 times higher than conventional PCDs, and mechanisms of deformation were completely different and were dominated by nano-twinning instead of by classic dislocation slip.

Cutting and abrasive properties have shown that NPDs always offer better wear resistance than SC or PDCs. For instance, the wear rate decreases from 200–1000 μm/m to 50–500 μm/m for PCDs and SC, respectively, and down to 10 μm/m for NPDs [128]. As another example, the flank wear of NPDs is reduced by a factor of 2–3 compared to PCDs, and by a factor 20 compared to SC [129,130] (Table 3).

Fracture toughness of MPD has been determined to be higher than the one of SC, and the wear/abrasion resistance to be 2 to 3 times higher than the best PDC [103].

- Optical properties

Optical properties of NPDs were also extensively studied using IR, UV and photoluminescence spectroscopy [128,133]. By combining these techniques, structural defects were revealed, such as the presence of nitrogen, small abundance of lonsdaleite, presence of a few dislocations and the absence of residual strain. Moreover, NPDs have larger bandgap than that of single-crystal diamond [193].

- Uses

Prior to considering these uses of NPDs, a great instrumental effort was made to be able to fabricate large pieces of ~1 cm^3^ [102]. This was possible due to the development of a 6000-ton Kawai-type multi anvil, as well as the selection of adapted grade of WC anvils and heater elements (Figure 7c). Thus, with such high-load apparatus, very high pressure was attainable even for very large volume samples.

Due to the exceptional properties of NPDs, they have potential applications in various industrial fields (cutting, drilling and electronics) and in new technologies. However, the industrial transfer for the fabrication of these NPDs seems extremely difficult. Indeed, the extreme high conditions of pressure and temperature (20 GPa, T > 2000 °C) needed for such synthesis require very high-pressure apparatuses that are very expensive (~1 M$) and very different from classic Toroid press usually used at much lower conditions (P < 8 GPa, T < 2000 °C). Moreover, these very high-pressure apparatuses are not easy to install and not easy to use, with a high risk of failure (so called blow-out).

Therefore, the main use for this NPD is limited to scientific fields, especially in high pressure research, either for their mechanical or optical properties. A Special Issue was published recently in High-Pressure Research (2020) in order to provide the main uses of NPDs on different scientific topics. A brief description is given below.

Mostly, NPDs are used in diamond anvil cell (DAC) combined with X-ray spectroscopic techniques such as XAS and EXAFS. Indeed, NPDs anvils have the advantage to produce a weak background in the signal and to not produce any glitch in the signal compared to conventional single-crystal anvils [194,195,196,197,198]. Thus, very fine local structure in the samples can be determined over a wide range of pressure. Similarly, NPDs were also used as anvils for neutron diffraction studies [199,200]. These recent developments have shown that pressure up to 80 GPa can be reached with a very “clean” neutron diffraction pattern, making NPDs anvils suitable materials for in situ studies of low Z materials at high pressure.

NPDs are also used to go further under high pressure combined with these spectroscopic techniques. Double stage DAC (ds-DAC) was also used with NPDs balls as double stage anvils, as first proposed by [143]. According to the shape and size of the double stage, pressure up to 400 GPa has been reached with a still acceptable XAS signal [201,202]. Concerning this ds-DAC set-up, there is still a debate on the value of the high pressures reached, basically due to pressure scaling problems at Mbar [203]. Again, in the same idea, in Paris–Edinburgh press (PE), NPDs were used as a new opposed type double stage to reach pressure up to 130 GPa [204]. The NPD double stage was inserted into the first stage of PE. With this method, sample size could be 100 times bigger than corresponding pressure obtained using conventional DAC. However, no temperature test was performed, making this set-up relatively limited for Earth sciences purposes.

Optical properties were investigated and have shown that NPDs are suitable as pressure and hydrostaticity/isostaticity sensors in high-pressure apparatuses combined with X-Ray/neutron sources [205].

## 7. Summary and Outlook

Diamond synthesis has always fascinated scientists since the beginning of the last century. A lot of different techniques were developed to mimic natural conditions of diamond formation (Figure 2, Figure 5, Table 4). Basically, there are five main different ways to synthesize/sinter diamonds that cover wide ranges of pressure and temperature, typically from few Pa (in CVD) to several hundreds of GPa (in shock) and from 800–1000 °C (in hydrothermal) to 2000–2500 °C (in HPHT) (Table 4). Each of these techniques has their own advantages and disadvantages based on their P–T conditions, field of applications, technical difficulties and industrial transfer capabilities. From a synthesis point of view, all these five techniques are able to fabricate diamonds, but the yield is very variable, from a few percent (in hydrothermal and SPS) to fully transformed samples (in CVD, HPHT or shock). Moreover, grain size produced is highly variable, from nanometer- to several-hundred-micrometer grains, according to the different techniques, and even within the same techniques but using different P, T and t conditions. Among these techniques, only two are able to sinter diamonds to objects of few mm^2^ (in CVD and HPHT), up to 1 cm^2^ (in HPHT), and to single-crystal or polycrystalline objects. These techniques are CVD and HPHT (Table 4), whereas SPS, hydrothermal or shock produces isolated grains, as powder, with a wide range of grain sizes, from nm to several hundred μm.

In terms of applications, diamond powders are mainly devoted to abrasives whereas compact objects have many more application fields. In detail, due to their smaller sizes and higher purity, CVD diamonds are used as optic windows or thin tips on cutting tools, whereas HPHT diamonds are used as cutting tools for building or drillings.

Finally, among all of these techniques, HP–HT process is the most used and has experienced the most developments since the mid-1950s, and the first synthetic diamonds. Indeed, diamond objects were, for decades, synthesized and sintered using binders (called PCD), in metallic matrix for the synthesis and using mostly cobalt for the sintering, in order to decrease P–T conditions of formations to few GPa (4–5) and medium temperature (1400–1500 °C) suitable for industrial transfer. This process is able to produce objects with mechanical properties adapted to cutting and drilling, but their duration is limited (hardness only 70 GPa and thermal stability up to 400 °C, a temperature easily reached in drilling due to friction).

Today and after two decades of technical developments using multi-anvil press, HP–HT process is able to synthesize and sinter diamond bodies (up to 1 cm^2^) without any binder at 20–25 GPa and 2000–2500 °C. This process, called direct conversion sintering (DCS), produces polycrystalline diamonds (mostly with nanometer grain size) that have exceptional mechanical properties, even better than single-crystal diamonds. A lot of work has been made to constrain mechanisms of this DCS as well as to improve the quality of the final product. However, an industrial transfer seems difficult as pressure and temperature remain very high, such that apparatuses are expensive and not easy to use. The next step would be therefore to lower conditions of sintering and keeping exceptional properties.

Indeed, as shown in the phase diagram of carbon (Figure 5), there is an important gap between the synthesis/sintering of diamonds using CVD process (low-pressure–medium-temperature, LP–MT) and those sintered by multi-anvil press (HPHT). Thus, preliminary works were made on the direct sintering of diamond objects from micrometer-grain-size diamond powders, either natural or synthetic (e.g., [131,134]). In this way, the energy delivered by high pressure and high temperature is only devoted to the formation of grain boundaries instead of graphite-to-diamond transition and formation of grain boundaries in the DCS process. Encouraging results show that diamond objects could be obtained at pressure around 12–15 GPa and 2000 °C, still far from conditions achievable in the industry.

The future challenges would be therefore to discover a way or different ways to lower these conditions of synthesis/sintering binderless diamonds compact. Indeed, even though the phase diagram of C is relatively well known and well constrained in terms of P–T stability fields, others parameters could be crucial to reach more “metastable conditions” at which the procedures of synthesis and/or sintering would be efficient. For example, heating and cooling rates, compression and decompression rates, control of grain size and distribution, control of hydrostaticity/isostaticity during the cold compression, and purity of precursors are among the possibilities to explore. Among these potential parameters to tune with, grain size seems to be of primary importance. Indeed, the reactivity of powder is extremely sensitive to the average grain size, its distribution and its purity. For example, it is known that the maximum packing of spheres is only ~75%; therefore, mixing powders with different grain sizes would help to compact the powder with limiting grain fragmentation and high level of stress during cold compression [131,206]. These potential damages have to be repaired at high temperatures, demanding higher conditions of sintering. It can be concluded from the present literature review that high-pressure–high-temperature conditions have high potential in yielding diamonds.

Finally, analytical progress should be made to characterize the mechanical properties of such hard materials, e.g., the development of non-destructive methods. Finally, the debate on lonsdaleite as a recoverable phase and on its potential higher mechanical properties than diamonds should be continued.

## Figures and Tables

**Table 1:** Lattice parameter of the three natural forms of carbon.

	Occurrence	Lattice	Space Group	Unit Cell (Å)	Density (g/cm^3^)	Hybridization
Graphite	Ambient conditions	Hexagonal	(194) *P*6_3_/*mmc*	a = b = 2.456 c = 6.696 ^1^	2.26	sp2
Lonsdaleite	HPHT	Hexagonal	(194) *P*6_3_/*mmc*	a = b = 2.51 c = 4.12 ^2^	^4^ 3.30–^5^ 3.52	sp3
Diamond	HPHT	Cubic	(227) $Fd\bar{3}m$	a = 3.56679 ^3^	3.51	sp3

^1,3^ from [6] ^2^ from [7] ^4^, measured ^5^, calculated.

**Table 2:** Experimental conditions for the synthesis and/or sintering of binderless diamonds.

Study	Apparatus	Starting Material (SM)			Pressure (GPa)	Temperature (°C)	Time (Min)	Yield (%)	Remarks
		Phase	Type	Grains Size (μm)					
Bundy, 1963	Belt-type press	Gr, SC, Gr, aC	R	-	11.8–15	up to 3900	~10^−7^	-	1st direct transition reported
Naka et al., 1976	-	Gr	R	-	14	up to 3500	4	60	10–20 μm diamond grain recovered
Iriune et al., 2003	Kawai type MAA	Gr	R	-	12–25	2300–2500	-	up to 100	10–200 nm grain size in NPD
Davydov et al., 2004	Toroïd Press	PAH	P	-	8	1500	1	60	5–40 μm monocrystals, 100% yield for T > 1350 °C, and graphitization step
Sumiya et al., 2004	Kawai type MAA	Gr	R	-	18	2300–2700	0.2–170	100	Microstructure: homogenous fine structure + lamellar structure
Dubrovinskaia et al., 2005	Kawai type MAA	C60	P	-	20	2200	-	100	Diamonds nanorods aggregated 5–20 nm Ø, >1 mm length, compact body of 1.8 mm Ø and 3 mm high
Dubrovinskaia et al., 2005	Kawai type MAA	C60	P	-	13–20	27–2127	<90	up to 100	5–12 nm grain size of diamond, lonsdaleite often present
		aC			20	2027			
		Gr			20–25	2177–2327			
		D			20	2027			
Le Guillou et al., 20,007	Kawai type MAA	QAS	-	-	15	1500	15–60	up to 100	Transforamtion path highly dependent on the nature of the precursors, especially their crystallinity. Lonsdaleite can be present
		CB		30–100 × 10^−3^		1500–1700–1900			
		HTCB		30–100 × 10^−3^		1500–1700			
		pGr		0.5–1		1500–1700–1900			
		HOPG		>1		1700			
Sumiya and Irifune, 2007, 2008	Kawai type MAA	Gr	R	-	15–28	2300–2500	0.2–10	100	Lower hardness for other C precursors than graphite
		aC	P	-	18–21	1800–2000	10–20		
		GC	P	-	18–21	2000–22,500	6–20		
		C60	P	-	18	1800–2000	30–35		
Merlen et al., 2009	DAC, PE, MAA	SWCNT +/− I	P	-	12–25	25–1527	60–120	up to 100	Graphitization step
Isobe et al., 2010	Kawai type MAA	Gr	R	-	12–14	2200–2600	5–30	up to 100	Minimum condition for NPD. Higher pressure means lower T to complete transformation.
Couvy et al., 2011	Kawai type MAA	Gr	R	-	19	2400	0.5	100	-
Dubrovinsky et al., 2012	Kawai type MAA	GC	B	20–50	20	1927	<15	100	Nano polycrystalline (50 nm) balls of 15–40 microns, presence of NaCl or Na2CO3 to make microballs.
He et al., 2013	Hinge-type cubic press	μD	P	-	8–20	1400–2500	1–30	no need	-
Xu et al., 2013	Hinge-type cubic press	Gr +/− milled	P	5–20	16	2500	1–10	100	Reaction at lower T with ball-milled Gr
Chang et al., 2014	Kawai type MAA	Gr	R	-	15	2327	20	100	NPD shaped in anvil, cyliner or sphere
Solopova et al., 2015	Kawai type MAA	GC	B	20–50	18	1850–2000	1–5	up to 100	Nano polycrystalline balls of 15–40 microns, presence of NaCl or Na2CO3 to make microballs
Lu et al., 2017	Hinge-type cubic press	μD	P	0.5	14	2000	1	no need	Cylinder of 3 mm Ø & high at the end. Some backtransformation in graphite. Final grain size of 170 nm
Liu et al., 2018	Hinge-type cubic press	μD	P	8–12	14	1000–2000	-	no need	-
Zhan et al., 2020	Hinge-type cubic press	μD	P	8–12	14	up to 1900	-	no need	Cylinder 11 mm Ø, 6 mm high. Not transparent at all. There may be pores and/or cracks
Li et al., 2020	Hinge-type cubic press	μD	P	8–12	16	2300	10	no need	Same size body as above, 10-micron final grain size
Osipov et al., 2020	Toroïd press	nD	P	25 × 10^−3^	7	1300	0.2	no need	Purification + sintering using ethanol at HPHT
Kawamura et al., 2020	Kawai type MAA	C_18_H_36_O_2_	P	-	10–13-17	600–1600	5–180	up to 100	Graphitization step, 10 nm grain at the end, control fO2 in some exp and remove water during exp
Park et al., 2020	DAC	C_10_H_16_,C_14_H_20_,C_18_H_24_	P	0.5–2 × 10^−3^	5–22	627–2727	~10^−7^	up to 100	Lower transition than with pure C source. Direct transformation, no graphitization step
Irifune et al., 2020	Kawai type MAA	GC	R	-	15–25	1700–2300	20	up to 100	Grain size dependence on P,T. P,T limits to keep nanograin

Gr = graphite, SCGr = single-crystal graphite, PAH = PolyAromatic Hydrocarbon, aC = amorphours Carbon, D = diamond, QAS = quasi amorphous carbon, CB = carbon black, HTCB = high-temperature carbon black, pGr = pyrolythic Graphite, HOPG = highly orientated pyrolythic graphite, SWCNT = single-wall carbon nano tube, μD = microdiamond, nD = nanodiamond, R = rods, P = powder, and B = balls. MAA = multi-anvil apparatus.

**Table 3:** Common physical properties of diamond.

Mechanical Properties	
Poisson’s ratio	0.1
Young’s modulus (GPa)	1050
Fracture toughness K1C (MPa.m^0.5^)	
Natural single crystal	5
Synthetic polycrystalline (NPDs)	8.5
Fracture strength (GPa)	
Natural single crystal	2.5–3.0
Synthetic polycrystalline (NPDs)	0.2 to 1.1
Transverse rupture strength (GPa)	
Natural single crystal	1.0–2.0
Synthetic polycrystalline (binder)	2.5 up to 400 °C, 0.5 at 500 °C
Synthetic polycrystalline (NPDs)	3.0 up to 1000 °C
Flank wear width/cutting length (μm/m)	
Natural single crystal	11.25
Synthetic polycrystalline (binder)	0.50
Synthetic polycrystalline (NPDs)	0.16
Knoop hardness (GPa)	
Natural single crystal	70 to 120 according to orientation
Synthetic polycrystalline (binder)	50–80 according to binder content
Synthetic polycrystalline (binderless)	
From graphite or diamond	125–145
From other C-precursors	80–100
Friction coefficient (μ)	0.05 to 0.15 according to orientation
**Optical properties**	
Color (see type classification)	colorless to various
Transparency	(X-ray) UV to IR
Absorption	2.5–6 μm
Fluorescence	UV
Refractive index	2.417 (0.044 dispersion)
**Electrical properties**	
Conductivity (Ω.m)	
Most natural diamonds	Insulator, 10^11^ to 10^18^
Type IIb (boron-doped, natural and synthetic)	Semiconductor, superconductor at 4 K
Electronic gap	5.5 ev
Dielectric constant	5.58 at 35 GHz
**Thermal properties**	
Expansion coefficient (10^−6^ × K^−1^)	1.0 at 600 °C, 4.4 at 1300 °C
Conductivity (W/(m.K))	
Natural single crystal	2200
Synthetic (^12^C enriched)	up to 3200
Stability at 1 bar	
	700 °C in air
	up to 1600 °C in Ar

**Table 4:** Summary of P–T conditions of the different techniques to synthesize and/or sinter diamonds.

Techniques	Pressure (GPa)	Temperature (°C)	Synthesis	Yield	Sintering	Size of Sintered Objects
**CVD**	10^−8^–10^−9^	800–1500	✓	very high	✓	few mm^3^
**SPS**	0.1	1500–1600	✓	low	✕	-
**Hydrothermal**						
LP-LT(MT)	0.1–2	300–1200	✓	very low	✕	-
HP–HT(MT)	3–8	1000–2000	✓	low	✕	-
**HPHT**			✓			
Catalyst	4–5	1400–1500	✓	high	✓	cm^3^
Binderless	15–25	2000–2500	✓	very high	✓	cm^3^
**Shock**	>30	>2000	✓	very high	✕	-

## Data Availability

Data sharing is not applicable in this article (no new data were created or analyzes in this study).
